# Systematically testing human HMBS missense variants to reveal mechanism and pathogenic variation

**DOI:** 10.1016/j.ajhg.2023.08.012

**Published:** 2023-09-19

**Authors:** Warren van Loggerenberg, Shahin Sowlati-Hashjin, Jochen Weile, Rayna Hamilton, Aditya Chawla, Dayag Sheykhkarimli, Marinella Gebbia, Nishka Kishore, Laure Frésard, Sami Mustajoki, Elena Pischik, Elena Di Pierro, Michela Barbaro, Ylva Floderus, Caroline Schmitt, Laurent Gouya, Alexandre Colavin, Robert Nussbaum, Edith C.H. Friesema, Raili Kauppinen, Jordi To-Figueras, Aasne K. Aarsand, Robert J. Desnick, Michael Garton, Frederick P. Roth

**Affiliations:** 1 Donnelly Centre, University of Toronto, Toronto, ON M5S 3E1, Canada; 2 Department of Molecular Genetics, University of Toronto, Toronto, ON M5S 1A8, Canada; 3 Lunenfeld-Tanenbaum Research Institute, Sinai Health, Toronto, ON M5G 1X5, Canada; 4 Department of Computer Science, University of Toronto, Toronto, ON M5S 2E4, Canada; 5 Institute Biomedical Engineering, University of Toronto, Toronto, ON M5S 3G9, Canada; 6 Advanced Academic Programs, Johns Hopkins University, Washington, DC 20036, USA; 7 Invitae Corp, San Francisco, CA 94103, USA; 8 Research Program in Molecular Medicine, Biomedicum-Helsinki, University of Helsinki, 00290 Helsinki, Finland; 9 Fondazione IRCCS Ca' Granda Ospedale Maggiore Policlinico, Unit of Medicine and Metabolic Diseases, 20122 Milano, Italy; 10 Porphyria Centre Sweden, Centre for Inherited Metabolic Diseases, Karolinska Institutet, Karolinska University Hospital, 17176 Stockholm, Sweden; 11 Centre français des porphyries, hôpital Louis-Mourier, Assistance Publique-Hopitaux de Paris, 92701 Colombes, France; 12 Centre de recherche sur l’inflammation, Université Paris Cité, UMR1149 INSERM, 75018 Paris, France; 13 Porphyria Expertcenter Rotterdam, Center for Lysosomal and Metabolic Diseases, Department of Internal Medicine, Erasmus MC, 3015 Rotterdam, the Netherlands; 14 Biochemistry and Molecular Genetics Department, Hospital Clínic, IDIBAPS, University of Barcelona, 08036 Barcelona, Spain; 15 Norwegian Porphyria Centre, Department of Medical Biochemistry and Pharmacology, Haukeland University Hospital, 5021 Bergen, Norway; 16 Department of Genetics and Genomic Sciences, Icahn School of Medicine at Mount Sinai, New York, NY 10029, USA

**Keywords:** HMBS,, hydroxymethylbilane synthase, acute intermittent porphryia, AIP, acute hepatic porphryia, variant effect mapping, deep mutational scanning, molecular dynamics, clinical variant interpretation, heme biosynthesis

## Abstract

Defects in hydroxymethylbilane synthase (HMBS) can cause acute intermittent porphyria (AIP), an acute neurological disease. Although sequencing-based diagnosis can be definitive, ∼⅓ of clinical HMBS variants are missense variants, and most clinically reported HMBS missense variants are designated as “variants of uncertain significance” (VUSs). Using saturation mutagenesis, *en masse* selection, and sequencing, we applied a multiplexed validated assay to both the erythroid-specific and ubiquitous isoforms of HMBS, obtaining confident functional impact scores for >84% of all possible amino acid substitutions. The resulting variant effect maps generally agreed with biochemical expectations and provide further evidence that HMBS can function as a monomer. Additionally, the maps implicated specific residues as having roles in active site dynamics, which was further supported by molecular dynamics simulations. Most importantly, these maps can help discriminate pathogenic from benign HMBS variants, proactively providing evidence even for yet-to-be-observed clinical missense variants.

## Teaser

van Loggerenberg et al. functionally assay ∼90% of all possible missense variants in *HMBS*, which is associated with acute intermittent porphyria. The resulting variant effect maps reveal sequence-structure-function relationships and provide evidence to distinguish pathogenic from benign variants.

## Introduction

Acute intermittent porphyria (AIP; MIM: 176000) is caused by deficiency in the heme biosynthetic enzyme hydroxymethylbilane synthase (HMBS; MIM: 609806, EC 2.5.1.61; also known as porphobilinogen deaminase [PBGD]).^1^ AIP, the most frequent acute hepatic porphyria, is an autosomal dominant disorder with an estimated prevalence of 1 in ∼1,700.^2^ The clinical penetrance of AIP is low (1%–38%), and ∼65% of AIP heterozygotes remain asymptomatic (i.e., have “latent AIP”) throughout their lives.^2^^,^^3^^,^^4^ AIP is characterized by potentially life-threatening acute attacks, precipitated by genetic and environmental factors that induce aminolevulinate synthase 1 (ALAS1; MIM: 125290), the first and rate-limiting enzyme in heme synthesis. *HMBS* encodes both erythroid-specific and ubiquitous (housekeeping) isozymes, differing in that the erythroid isoform has a later translational start site that eliminates 17 amino acids at the N-terminus.^5^^,^^6^ In classical AIP, both erythroid-specific and ubiquitous HMBS isoforms are deficient. However, ∼5% of AIP cases are non-erythroid, caused by a variant that affects only the ubiquitous isoform of HMBS.^7^ In cases of increased heme demand in the liver, the combination of ALAS1 induction and HMBS deficiency results in the accumulation of the porphyrin precursor porphobilinogen (PBG), as well as δ-aminolevulinic acid (ALA),^1^^,^^8^ which is likely neurotoxic.

The fact that AIP is a rare disorder, with attacks that are typically episodic and have nonspecific neurovisceral symptoms, can delay clinical recognition and intervention.^3^^,^^9^^,^^10^ In a symptomatic individual, biochemical diagnosis of AIP is based on demonstrating highly elevated plasma or urine levels of ALA and PBG, after excluding other acute porphyrias by analysis of porphyrin markers in urine and feces. Where a suspected AIP attack is not reported to clinicians in a timely fashion, however, biochemical diagnosis may be more complicated. Both in the latter scenario and more generally, sequencing the three acute porphyria genes (*HMBS, CPOX* [MIM: 612732], and *PPOX* [MIM: 600923]) to identify a causative variant in an individual with an acute porphyria can be useful. Sequencing has further utility in confirming diagnoses and identifying the *HMBS* variant causing AIP, which facilitates screening of healthy family members to identify individuals who are at-risk for AIP (i.e., have latent AIP). Those identified with latent AIP can then be recommended lifestyle and medication measures to reduce the risk of acute attacks and be monitored for development of long-term complications such as primary liver cancer.^11^ In recent years, HMBS has been included in gene panels both for inborn error of metabolism and familial liver cancer and has also been suggested to be a tumor-suppressor gene.^12^

Out of the 472 clinical HMBS variants reported in ClinVar, 166 (35%) have been annotated as a “variant of uncertain significance” (VUSs), and most (69%) of these VUSs are missense variants.^13^ The increasing role of sequencing-based diagnostics underlines the importance of providing better tools for variant classification, especially for missense variants. Functional assays can provide strong evidence for clinical variant interpretation, but where they are performed at all, they are typically resource intensive. Moreover, functional assays are typically “reactive,” performed only after (and often years after) the first clinical presentation of a variant. By contrast, computational methods can predict the impact of all missense variants “proactively,” in advance of the first clinical presentation. Although computational predictors are steadily improving,^14^ this type of evidence is considered weak at best under current American College of Medical Genetics and Genomics and Association for Molecular Pathology (ACMG/AMP) guidelines.^15^

The functional impact of essentially all possible single variants in a given target protein can be revealed by multiplexed assays of variant effect.^16^ Variant effect maps can provide accurate and proactive identification of dysfunctional alleles^17^^,^^18^; for example, analysis of variant effect maps for three cancer-related genes^19^^,^^20^^,^^21^^,^^22^^,^^23^ yielded reclassification for 15%–69% of clinical missense VUSs.^17^

Model organism assays, e.g., measuring the ability of a human protein variant to complement loss of the corresponding ortholog’s activity in that model organism, can enable inference of human variant pathogenicity.^24^^,^^25^^,^^26^ Here, we employ a multiplexed yeast-based assay of the human HMBS to proactively and systematically measure missense variant impacts for both erythroid and ubiquitous HMBS isoforms. We find that the resulting impact scores correspond well with prior knowledge about the atomic structure of human HMBS and with known patterns of mutational tolerance. We use the map, together with molecular dynamics (MD) simulations, to implicate residues in the control of backbone flexibility and in motions of a “lid” over the active site. Finally, we demonstrate that variant effect map scores can reliably identify pathogenic HMBS alleles.

## Material and methods

### Strains and plasmids

The *Saccharomyces cerevisiae* strain with which we assayed *HMBS* variant libraries (*MATα ts-hem3::KanR his3Δ1 leu2Δ0 ura3Δ0*) was kindly provided by Drs. Guihong Tan, Charles Boone, and Brenda Andrews. For yeast expression, we used the Gateway-compatible destination vector pHYC-Dest2 (CEN/ARS-based, *ADH1*promoter, and *LEU2* marker).^24^ The *HMBS* open reading frame (ORF) clone (Ensembl: ENST00000652429.1, GenBank: NM_000190.4) was obtained from the Human ORFeome v.8.1 library.^27^

Wild-type (WT) reference or mutated disease-associated versions of the HMBS ORFs were transferred into pHYCDest by Gateway LR reactions. After confirmation of ORF identity and expected mutations by Sanger sequencing, the expression clones were transformed into the appropriate yeast temperature-sensitive (ts) strain in parallel with an “empty” expression vector control (bearing the counterselectable ccdB marker controlled by a bacterial promoter).

### HMBS assay validation

For yeast ts mutants transformed with vectors expressing HMBS cDNAs, cells were grown to saturation at 30°C. Each culture was then adjusted to an optical density at 600 nm (OD_600_) of 1.0 and serially diluted by factors of 5^−1^, 5^−2^, 5^−3^, 5^−4^, and 5^−5^. These cultures (5 μL of each) were then spotted on SC-LEU plates as appropriate to maintain the plasmid and incubated at either 30°C or 35°C for 48 h. After imaging, results were interpreted by comparing the growth difference between the yeast strains expressing human genes and the corresponding empty vector control (Figure S1). Two independent cultures were grown and assayed.

### Constructing codon-randomized HMBS variant libraries

Libraries of *HMBS* variants were constructed with an oligo-directed codon-randomizing mutagenesis (precision oligo-pool-based code alteration or POPCode) method.^28^ Mutagenesis was targeted to each of two equal-length regions so that two full-length mutagenized libraries were generated for each HMBS isoform. Briefly**,** we designed a ∼35 nt oligonucleotide corresponding to each codon along the entire length of the *HMBS* ORF, each with a central NNK degeneracy targeting that codon for randomization, by using the POPcode oligo suite tool.^28^ From the 360 oligos synthesized, oligos for each region were combined to produce two regional pools and then phosphorylated. For each isoform, uracilated full-length WT *HMBS* was used as the template and two separate annealing reactions (with Kapa HiFi Uracil+ DNA polymerase (KapaBiosystems) and a dNTP/dUTP mix) were set up with either oligo pool. After annealing phosphorylated oligos, KAPA HiFi Uracil+ DNA polymerase (Kapa Biosystems) was used to fill in gaps and Taq DNA ligase (NEB) was applied to seal the nicks. Treatment with Uracil-DNA-glycosylase (UDG) degraded the original uracilated template, and the newly synthesized mutagenized strand was amplified with primers containing attB sites. The mutagenized attB-PCR products were then transferred *en masse* into the entry vector pDONR223 using Gateway BP reactions. These Gateway-entry clone libraries were transferred to a pHYC-Dest2 expression vector via *en masse* Gateway LR reactions to enable yeast expression. Both Gateway-entry and -host libraries were transformed into NEB 5-alpha *Escherichia coli* cells (NEB) and selected on LB agar plates containing spectinomycin and ampicillin, respectively. Next, host libraries were transformed into the *S. cerevisiae hem3-ts* mutant strain via the EZ Kit Yeast Transformation kit (Zymo Research). To retain high library complexity, plasmids were purified from >350,000 clones at each transfer step and ∼1,000,000 yeast transformants were pooled to form the host library.

### Multiplexed assay for HMBS variant function

The underlying yeast-based functional complementation assay of HMBS was previously established.^29^ High-throughput complementation screening was carried out as follows. Yeast transformants were grown at 30°C in synthetic complete (SC) media with glucose as carbon source, without leucine (SC-LEU; USBiological) to ensure plasmid retention (the non-selective condition). For each region, two plasmid pools were prepared from 10 optical density units (ODU) of cells (defined here as the number of yeast cells found in 10 mL of a 1 OD_600_ culture, which is typically ∼10^8^ cells) and used as templates for the downstream tiling PCR. Preserving genotype-phenotype linkage in a heterogeneous culture is challenged by the potential for diffusion of porphyrin precursors from phenotypically WT cells to HMBS-deficient cells. To limit these cell-non-autonomous effects on our multiplexed functional analysis of HMBS variants, two replicates of ∼800,000 cells from each of the regional transformant pools were washed three times to remove exogenous porphyrins and heme, plated on solid selective SC-LEU media (such that the initially seeded cells are likely to be isolated from one another), and grown at restrictive temperature (selective condition; 35°C) for 48 h. After pooling the colonies of each replicate, plasmids were extracted from 10 ODU of cells and used as template for downstream tiling PCR. In parallel, the *hem3-ts* mutant strain was transformed with the WT ORF and grown alongside the regional pools, and the plasmid was extracted from two of 10 ODU of cells for each condition for use as a control.

### Scoring functional impact of variants with TileSeq

Measuring variant effects with a pooled *en masse* selection strategy was done according to the previously described TileSeq approach.^28^ Briefly, for each of the plasmid libraries from non-selective and selective pools, short template amplicons (∼150 bp) that tile the ORF (within the context of each regional pool) were amplified with primers carrying a binding site for Illumina sequencing adaptors. Both regional pools consisted of five tiles. In the second-round PCR, Illumina sequencing adaptors with index tags were added to the first-step amplicons. Paired-end sequencing was then performed on the tiled regions across the ORF, thus dramatically reducing base-calling error and enabling accurate detection of very low (parts-per-million) variant frequencies. Separate sequencing runs performed for each isoform with an Illumina NextSeq 500 via a NextSeq 500/550 High Output Kit v.2 achieved an average sequencing depth of ∼2,000,000 paired-end reads per tile.

Sequencing reads were demultiplexed with bcl2fastq v.2.17 (Illumina) and processed with the previously described TileSeq strategy.^28^ In brief, variant frequencies in each condition were determined with Python scripts (https://github.com/RyogaLi/tileseq_mutcount, see web resources), which incorporated Bowtie2^30^ as part of the pipeline to align the sequence of each read pair to the reference template. Following alignment, variants were called when Q scores (based on both reads in the pair) indicated the variant to have a posterior probability exceeding 90%. Read counts were then normalized on the basis of sequencing depth to yield variant frequency data for each condition and replicate.

Processing of raw read count data was carried out with the R package tileseqMave (https://github.com/jweile/tileseqMave; see web resources).^26^^,^^28^ Briefly, to account for PCR and sequencing error and the possibility of a bottleneck effect from non-selective pool sampling, variants for which the number of read counts within the selective or non-selective library fell below a chosen threshold (10) or for which the frequencies fell below a custom percentile (90th) of variant frequencies observed in the WT library were filtered out. The effect of PCR and sequencing errors were reduced by subtracting WT from variant frequencies in both the non-selective and selective pools. Next, an enrichment ratio (Φ) was calculated for each variant with the ratio of adjusted frequencies in the selective to the non-selective library. A functional impact score (FS_MUT_) for each variant was calculated as ln(ΦMUT/ΦSTOP)/ln(ΦSYN/ΦSTOP), where ΦMUT is the enrichment ratio calculated for a given variant, ΦSTOP is the median enrichment ratio of all nonsense variants, and ΦSYN is the median enrichment ratio of all synonymous variants. Because truncations occurring close to the C terminus are less likely to be of functional significance, nonsense variants were excluded from the last 14 amino acids of each HMBS ORF for the purposes of calculating ΦSTOP. Functional impact scores of each isoform were first rescaled for each region separately, such that FS_MUT_ = 0 when ΦMUT = ΦSTOP and FS_MUT_ = 1 when ΦMUT = ΦSYN. However, differences in the stringency of selection for the two isoforms introduced non-linear changes in scale that differ between isoform maps. Scores for the erythroid isoform were therefore rescaled to minimize the average Euclidean distance between scores in the two maps. Filtering further for variants with high quality measurements, we removed variants that had a frequency below <0.005% in the corresponding non-selective library.

A “delta score” for each variant was calculated as the difference between functional impact scores for the two isoforms. To combine the variant effect maps where the score passed quality filtering in both isoforms, a simple weighted average was used: (FS_1_/σ^2^_1_+FS_2_/σ^2^_2_)/(1/σ^2^_1_ + 1/σ^2^_2_). Alternatively, the single available score was used when only one score was available. A combined estimate of measurement errors for scores in the combined map was derived by σ^2^ = 1/(1/(σ^2^_1_+σ^2^_2_)).

### Phylogenetic comparison of different models for hyper-complementation

To assess whether variants exhibiting "hyper-complementing," i.e., higher-than-WT, growth in the yeast complementation assay are likely to be advantageous, deleterious, or neutral in humans, we performed phylogenetic analysis as described previously.^28^^,^^31^ Briefly, we first normalized each variant’s score relative to the WT score for that position to avoid penalizing the WT variant itself in cases where its score is slightly greater than 1. Additional processing and imputation steps removed low-quality data and imputed likely values for missing data points, respectively. Three sets of scores (*s*) were generated from this dataset, each testing a different way of relating variant score for amino acid *a* at site *r* (*s*_*r*,*a*_) to the preference for amino acid *a* (defined as π_*r*,*a*_ = $\frac{s_{r,a}}{\Sigma_{a^{\prime }}s_{r,a^{\prime }}}$) across a set of aligned homologous sequences. In the first (“advantageous”) model, π_*r*,_ = *s*_*r*,*a*_. In the second (“neutral”) model, if *s*_*r*,_ > 1 then *s*_*r*,*a*_ = 1. In the third (“damaging”) model, if *s*_*r*,_ >1, we transformed it to a value of 1/*s*_*r*,*a*_, otherwise π_*r*,*a*_ = *s*_*r*,*a*_. For a set of 60 Ensembl homologs having at least 85% sequence identity to the human protein (Table S10), we applied the phydms software package (https://github.com/jbloomlab/phydms; see web resources) and determined the quality of fit to the phylogeny (as measured by the Akaike information criterion) under each of the three preference models.

### Reference set of disease- and non-disease-associated variants

To assess the ability of variant effect maps to identify pathogenic variants, we used a “positive” set of 53 variants identified as disease-associated collected by European expert centers. As a “negative” set, we used seven variants identified as non-disease-associated collected by European and American expert centers and four variants annotated as either benign or likely benign on ClinVar that had been submitted with review criteria and that did not have conflicting interpretations. We augmented the negative set with rare variants (MAF < 0.0005) from gnomAD v.2.1.1, requiring that they had been observed to be homozygous in at least one individual, and had no pathogenicity annotations. This identified an additional two “proxy-benign” variants, yielding a negative set with 13 variants total.

### Evaluating strength of evidence provided by functional impact scores for variant classification

To aid in clinical variant interpretation, we determined a quantitative Bayesian evidence weight for each variant within our maps by translating the scores to log likelihood ratios of pathogenicity (LLRps). To this end, we estimated the probability densities underlying the distributions of the scores of damaging and tolerated variants by kernel density estimation to obtain estimated probability density functions for pathogenic and benign variants: π + (s) and π − (s), respectively. The pathogenic:benign log likelihood ratio for a variant with a given functional impact score, s, was calculated as the ratio of the estimated probability density functions evaluated at s:

$$\log\left(\Lambda \right)=\log\;\left(\frac{P\left(s|Pathogenic\right)}{P\left(s|Benign\right)}\right)\approx \log\;\left(\frac{\pi_{+}\;\left(s\right)}{\pi_{-}\;\left(s\right)}\right)$$

A spreadsheet with all LLRp values can be found in Table S8. The code used for these analyses is located at https://github.com/jweile/maveLLR.

We next calibrated the relationship between LLR values and evidence strength categories within the ACMG/AMP framework, using an approach adapted from Tavtigian et al.^32^ Briefly, LLRs for descending evidence levels are modeled to decrease by a factor of 2 at each level starting from a fixed LLR, the "pathogenic very strong" (PVSt) level, which is chosen such that all ACMG/AMP evidence combination rulesets result in posterior probability of pathogenicity that satisfy the following constraints: >99% for all pathogenic (P) rules, >90% for all likely pathogenic (LP) rules, <10% for all likely benign (LB) rules, and <1% for all benign (B) rules. Here, we followed Tavtigian et al. in modeling a global prior probability of 0.1, as this enabled a set of LLR values for evidence at different evidence strength levels, which was internally consistent across the set of “path rules” established within the ACMG guidelines.^15^^,^^32^ The most conservative LLR thresholds fulfilling these constraints were found to be 2.54 for PVSt, 1.27 for pathogenic strong (PSt), 0.63 for pathogenic moderate (PM), 0.31 for pathogenic supporting (PSu), −0.31 for benign supporting (BSu), and −1.27 for benign strong (BSt).

### Computational details (MD simulation setup)

To examine variant-specific impacts on the dynamic behavior of HMBS and its movements during catalysis, we next performed MD simulations. Starting structures were obtained by applying necessary changes to the high-resolution X-ray crystal structure (Protein Data Bank [PDB] ID: 5M6R).^33^ Specifically, to study the apoenzyme system, the co-factor (DMP) and substrate (PBG units) were removed. Mutations were introduced by PyMol (The PyMOL molecular graphics system, version 1.3, Schrödinger, LLC.), and the protonation states of ionizable amino acids at pH = 7.4 were checked with H++ server.^34^

For simulations, we used AMBER parm14SB^35^ parameters and the TIP3P model for solvent.^36^ The nonstandard PBG structure was first energy minimized with the B3LYP DFT functional in combination with the 6-31G(d) basis set.^37^^,^^38^^,^^39^ Partial atomic charges were obtained from the electrostatic potential with the Gaussian 16 (Revision C.01).^40^ The remaining parameters were obtained from standard parm10 and GAFF2 parameter files, via the ANTECHAMBER module of the Amber18 software suite.^41^ All missing heavy atoms and hydrogen atoms were added with the LEaP module of Amber18.^41^

All systems were first neutralized with Na^+^ ions and solvated in an explicit TIP3P water box with at least 1.0 nm from the edge of the enzyme.^36^ Covalent bonds involving hydrogen atoms were constrained with the SHAKE algorithm^42^ and the particle mesh Ewald^43^^,^^44^ algorithm was used for long-range electrostatic interactions. The water molecules and ions were relaxed with 1,000 steps of steepest descent and 2,000 steps of conjugate gradient minimization, while the protein was constrained with a 500.0 kcal mol^−1^ Å^−2^ force constant. The entire system was then energy minimized with 1,000 steps of unrestrained steepest descent, followed by 2,500 steps of unrestrained conjugate gradient minimization. Subsequently, the system was heated from 0 to 300 K over 200 ps with restraints on the solute (10.0 kcal mol^−1^ Å^−2^). Each system was then equilibrated for 500 ps under a constant number of particles, volume, and temperature (NVT) condition. All simulations were carried out under a constant number of particles, pressure, and temperature (NPT) with Berendsen thermostat and barostat. Periodic boundary conditions were employed for all MD simulations, which were carried out for 500 ns for apoenzyme WT, p.Gly60Pro, p.Asp61Asn, p.Asp61Ala, and p.Phe77Ala mutants and for the p.Asp61Lys;Lys27Asp double-mutant. Simulations of 1 μs duration were performed for WT, p.Gly346Pro, and p.Glu250Arg mutant enzymes for their intermediate (ES2) enzyme-substrate conformations, which have two additional substrate pyrrole rings covalently bound to the dipyrromethane (DPM) cofactor.

System stability was measured throughout each simulation with root-mean-square deviations of backbone atoms (Table S2). Analyses were carried out with CC-PTRAJ of AMBER 18.^45^ Hierarchical agglomerative clustering was completed for the last 500 ns of the trajectories for ES2 systems on the basis of the conformations of DPM-PBG (substrate). The geometrical and interaction analyses were carried out for the structures in the highest occupied cluster. Hydrogen bonds were defined with a 120° angle (donor-hydrogen···acceptor) threshold and a 3.4 Å distance threshold between the donor and acceptor heavy atoms.

### Thermostability calculations

We calculated protein thermostability (ΔΔG) values to relate these to our map scores and thereby identified functionally important HMBS residue positions. Calculations of ΔΔG were carried out with DDGun3D version 0.0.2 (https://github.com/biofold/ddgun), as previously described.^46^ The PDB entry 3ECR for HMBS satisfied the following conditions: an X-ray determined structure with resolution 2.2 Å or better, monomeric structure, and no missing or non-standard residues.^47^ We defined stabilizing amino acid substitutions as those for which ΔΔG ≥ −1, and a destabilizing substitution for ΔΔG < −1.

### Comparison of functional impact scores and enzyme activity

Previous studies^2^^,^^4^^,^^48^^,^^49^^,^^50^^,^^51^ measured the enzymatic activity of HMBS variants expressed in *E. coli.* Of the 113 missense variants with measured activity, a subset of 102 that were also well measured in our combined map were used to investigate the relationship between our functional impact scores and enzyme activity (Table S7).

### Structure modeling and protein positional features

We used the Pymol software to place map scores in the context of a solved HMBS crystal structure (PDB ID: 5M6R). Using the InterfaceResidues.py script (https://pymolwiki.org/index.php/InterfaceResidues), we defined interfacial residues as a change in accessible surface area (ΔASA) greater than 1 Å^2^ between the complex and single-chain structures. We used the FreeSASA program (https://freesasa.github.io/) to calculate the relative solvent exposure of residue positions. After examining the distribution of relative solvent exposure values and it’s fit to a mixture of Gaussians (Figure S10), we established thresholds corresponding to the high and low peaks. Residues with surface area values exceeding 40% were considered exposed, while those below 20% were classified as buried.

### Normalization and transformation of computational predictor scores

To place scores (*s*_original_) from our combined map on a common scale with scores from the computational predictor ESM-1b,^52^ unity-based normalization was performed. In this normalization, 0 represents null-like variants, while 1 represents neutral variants. The scores were transformed with the following formula: *s*_tranformed_ = 1 − (*s*_original_ − min(*s*))/(max(*s*) − min(*s*)).

### Relating variant functional scores to population allele frequencies

Variants in HMBS were retrieved from UK Biobank (OQFE version of whole-exome VCF files; application ID: 51135), which includes sequencing data from ∼450,000 participants, as were non-overlapping gnomAD v.2 and v.3 datasets (https://gnomad.broadinstitute.org/). We then calculated odds ratios to evaluate the extent of allele depletion for variants with either damaging or neutral scores in our combined map.

## Results

### A scalable functional assay for HMBS missense variants

We implemented a scalable yeast-based functional complementation assay on the basis of the previous observations^29^ that a strain bearing a temperature-sensitive (ts) mutation in the essential yeast ortholog of *HMBS* (*HEM3*) exhibits reduced growth at the non-permissive temperature and human HMBS rescues this phenotype. The complementation relationship was confirmed (Figure S1; see material and methods), and we validated the assay (Figure S1) for an HMBS variant set that included four missense variants having a stringent “pathogenic” annotation in ClinVar and three “proxy-benign” (not known to be disease associated) missense variants having allele frequencies that roughly matched those of the pathogenic variants. To assess the functional impact of each variant in the yeast *hem3* ts strain, we again assessed growth at the non-permissive temperature (see material and methods). Each variant was assayed alongside strains with either the WT human protein or an empty vector, respectively serving as positive and negative controls for variant functionality. Yeast complementation assays for a variety of genes were previously shown to detect ∼60% of pathogenic variants at a stringency at which 90% of variants observed to be damaging were pathogenic (i.e., 60% recall at 90% precision).^24^ In line with this expectation, we observed 50% recall (lack of complementation for two of four pathogenic variants) with 100% precision (complementation for all non-pathogenic variants) (Figure S1).

### Systematic maps of HMBS missense variant functional impact

By coupling an efficient functional complementation assay with the TileSeq framework for multiplexed assays of variant effect, we sought to measure the functional consequences of all possible missense HMBS variants.^28^ First, we constructed libraries of HMBS variants (for both erythroid-specific and ubiquitous isoforms) by using our previously described POPCode mutagenesis method.^28^ To balance the objective of having substantial representation of each variant in the library against the objective of having roughly one amino acid substitution per clone, we generated two separate full-length libraries, with mutagenesis affecting the N- and C-terminal halves of the protein, respectively. Variant libraries were initially generated as a pool of amplicons. Large-scale sequencing showed that mutagenesis was relatively even across each of the libraries for both erythroid and ubiquitous HMBS isoforms, with an average of 1.7 amino acid changes per clone (Figure S2A). Amplicons were transferred *en masse* into the appropriate yeast expression vector via two steps of recombinational subcloning (see material and methods). The resulting mutagenized expression libraries were then transformed *en masse* into the appropriate ts yeast strain, yielding ∼2 million independent yeast transformants for each library.

**Figure 1:**
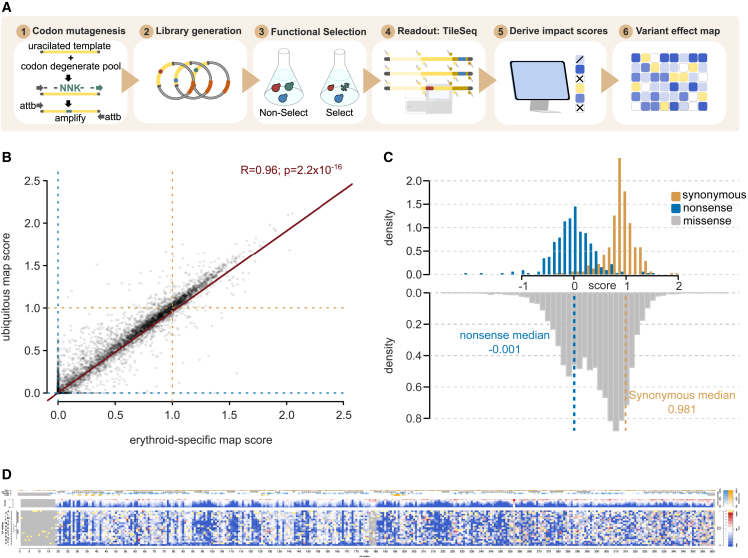
Generating and evaluating HMBS variant effect maps (A) Workflow for generating HMBS variant effect maps. (B) Correspondence between erythroid-specific and ubiquitous HMBS isoform functional scores. For reference, null- and WT-like scores are indicated with dashed blue or gold lines, respectively, while the red line corresponds to a linear regression fit (R = 0.96; p = 2.2 × 10^−16^). (C) Distributions of functional impact scores of nonsense (blue), synonymous (gold), and missense variants (gray) from the combined erythroid-specific and ubiquitous HMBS map. (D) Preview of full-sized combined HMBS map.

To assess the functional impact of many HMBS variants in parallel, pools of yeast HMBS mutant strains were grown competitively on synthetic medium at the non-permissive temperature. The frequencies of each HMBS variant within this laboratory strain population were then determined, before and after selection, with the TileSeq framework (Figure 1A).^28^ Briefly, we designed a set of amplicon “tiles” that collectively span the complete coding region. These tiles are sufficiently short (∼150 bp) to enable sequencing of both strands (“duplex sequencing”), with variants called only where they are detected on both strands. Each nucleotide position was covered by ∼2 million duplex reads. We considered variants appearing frequently enough in the pre-selection library (above 10 counts per million reads sequenced) to be well measured. This criterion was satisfied by >88% of all possible missense variants and by >95% of the amino acid substitutions that can be achieved via a single-nucleotide variant (SNV), for both the erythroid-specific and ubiquitous isoforms (Figure S2B). Comparing post-to pre-selection variant frequencies, we calculated a functional impact score (see material and methods), in both protein isoforms, for nearly all HMBS amino acid substitutions.

We estimated uncertainty (standard error) for each functional impact score both on the basis of agreement between two biological replicates and on trends in the behavior of replicates for other variants with similar pre-selection variant frequency (see material and methods).^28^^,^^53^ Impact scores with an estimated standard error above 0.3 were removed, yielding measurements of functional impact for >6,000 missense variants for each isoform. Thus, we obtained high-confidence functional impact scores for 87% and 84% of all possible amino acid substitutions in the erythroid and ubiquitous HMBS isoforms, respectively (Figure S2B). These included 93% (for the erythroid isoform) and 90% (for the ubiquitous isoform) of the amino acid substitutions accessible by a single-nucleotide change.

Impact scores for erythroid and ubiquitous maps were highly correlated (Pearson’s r = 0.96, Figure 1B). Indeed, where scores were available from both maps, no convincing difference between the maps was observed in any segment of HMBS (Figure S17B). We therefore calculated a weighted average score for each variant (see material and methods) to generate a single combined map. All scores for the erythroid, ubiquitous, and combined variant effect maps are publicly available (MaveDB^54^ accession urn:mavedb:00000108-a).

For each individual map, as well as the combined map, the impact score distributions of synonymous and nonsense variants were well separated (Figures S2C and 1C). Missense variants from each map showed a bimodal distribution, suggesting that variants tended to either have strong or neutral functional impacts as opposed to having intermediate effects. A small fraction (2.5%) of missense variants exhibited “hyper-complementation,” providing growth rescue beyond that of WT human HMBS in yeast.

### Hyper-complementing HMBS variants are likely deleterious in humans

It has been previously reported that, for SUMO and UBE2I (the human SUMO E2 conjugase), hyper-complementing variants displaying increased fitness in yeast assays may in fact be disadvantageous in humans.^28^ We explored this idea for HMBS by using a quantitative phylogenetic approach^55^^,^^56^ that compares three hypotheses about the effects of hyper-complementing variants: (1) variants that confer an advantage in our maps will also do so in humans and related species; (2) hyper-complementing variants are equal in function to WT; and (3) hyper-complementing variants are deleterious in humans and related species (a model in which the functional score in humans is modeled as the reciprocal of the observed score in yeast). We found the third (deleterious) model to be the best performing for HMBS (Table S1). We also compared our hyper-complementing map scores with scores from sequence-based computational predictor ESM-1b^52^ after placing them on a common scale (see material and methods), finding that ESM-1b showed more damaging scores for variants showing hyper-complementation than variants showing WT-like activity in our assay (Figure S3). Together these results argue that hyper-complementing variants in our yeast assay should be treated as deleterious in humans.

### Functional scores captured known roles for many HMBS missense variants

**Figure 2:**
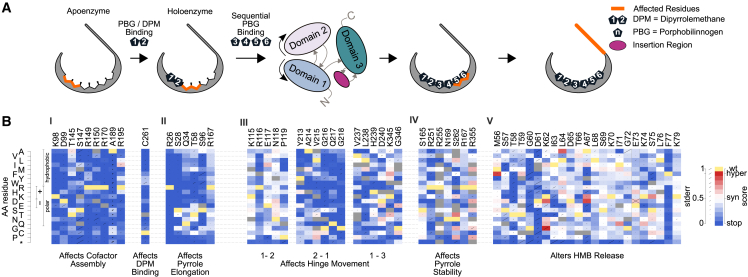
Identifying patterns of mutational tolerance (A) An overview of pyrrole-binding sites and structural fluctuations of HMBS involved in polypyrrole elongation and HMB formation. (B) Functional scores for each possible substituted amino acid (y axis) at each active-site residue position (x axis) responsible for (I) altering cofactor binding, (II) PBG binding for pyrrole chain elongation, (III) hinge flexibility, (IV) pyrrole stability, and (V) HMB release. For each substitution, diagonal bar sizes convey estimated measurement error in the corresponding functional score. Box color either indicates the WT residue (yellow); a substitution with damaging (blue), tolerated (white), or above-WT (“hyper-complementing,” red) functional score; or missing data (gray).

Several features of HMBS biochemistry were recapitulated in our variant effect maps. HMBS activity begins with condensation of two porphobilinogen (PBG) molecules to assemble DPM, to which four additional units of PBG are subsequently condensed (and then released by hydrolysis) to generate hydroxymethylbilane (HMB).^57^^,^^58^ Importantly, DPM is bound covalently at Cys261 and, as expected, our maps found this critical cysteine to be intolerant to mutation (Figure 2B, I).^59^

Several residues are known to be important for DPM assembly, enzyme kinetics, and conformational stability, including Lys98, Asp99, Thr145, Ser147, Arg149, Arg150, Arg173, and Arg195.^49^^,^^60^ Our maps found all of these critical positions to be generally intolerant to mutation (Figure 2B, I). Although one study suggested hydrogen-bonding of Ala189 to DPM,^49^ it has not been described as critical for function. Ala189 displayed intolerance to mutation in our map, suggesting that its role in stabilizing the polypyrrole chain may be more critical than previously recognized. Five additional active site residues involved in polypyrrole chain elongation—Arg26, Ser28, Gln34, Thr58, and Ser96—were each found to be important but slightly more tolerant of variation than those that interact with DPM (Figure 2B, II).^49^

Another six residues (Ser165, Asn169, Arg251, Arg255, Ser262, and Arg355) have been predicted to play a key role in stabilizing the growing pyrrole chain.^49^^,^^61^ Of these, our map surprisingly only implicated Arg251 and Ser262 as being essential for HMBS function (Figure 2B, IV). Also unexpectedly, we found Arg167, a residue reported to play a dual role in both catalysis and HMB release,^49^ to be highly tolerant to mutation (Figure 2B, IV). Arg167 is unquestionably important in humans,^2^ with five Arg167 missense variants having either “pathogenic” and/or “likely pathogenic” classification.^13^ Where a protein has multiple functions and only a subset of these are required to provide growth rescue in a complementation assay, the assay will only detect impacts of a variant on the subset of functions required for rescue. That we find Arg167 (as well as Ser165, Asn169, Arg255, and Arg355) to be tolerant to substitution could potentially be explained if the addition of the final two of six PBG monomers were not required to rescue the *hem3 ts* mutation in yeast. One scenario for this is that either (1) production of the tetrapyrrole form of HMB is sufficient to sustain growth of the yeast *hem3 ts* strain or (2) residual activity of the *HEM3* ts mutant can extend the tetrapyrrole form to generate the full hexapyrrole.

The active site loop (residues 56–76), with residues Gly60-Ile71 adopting α-helical secondary structure, is known to contribute to the recruitment of PBG and chain elongation.^61^ Within this loop, residues Thr58, Asp61, Ser69, and Lys70 have been noted as important by some studies, and other studies also implicate residues Lys74 and Lys79.^33^^,^^49^^,^^61^ Our data support an important role for Thr58, Gly60, and Asp61 residues in enzyme function (Figure 2B, V). However, we found that substitutions in residues Lys70, Lys74, and Lys79 previously reported as important for stabilizing DPM were generally tolerated (Figure 2B, V).^61^

### Functional impact scores point to key residues modulating HMBS structural fluctuations

The HMBS active site cleft is at the interface of domain 1 (residues 1–114, 219–236) and domain 2 (residues 120–212).^62^ Structural studies have suggested that movement of HMBS domains 1, 2, and 3 (residues 241–361), facilitated by flexible inter-domain hinge regions, helps accommodate substrates of various sizes during PBG chain elongation.^33^^,^^63^ (Here, we refer to: “hinge 1-2,” connecting the N-terminal segment of domain 1 with domain 2; “hinge 2-1,” connecting domain 2 with the C-terminal segment of domain 1; and “hinge 1-3,” connecting the C-terminal segment of domain 1 with domain 3.) Consistent with this model, our maps showed severe fitness defects for mutations affecting hinge 2-1 and hinge 1-3. In addition, Gly346, positioned within a “hinge 3-3” preceding the C-terminal helix in domain 3, is intolerant to variation (Figure 2B, III). Surprisingly however, we found hinge 1-2 residues to be highly tolerant to mutation (Figure 2B, III).

MD simulations have previously suggested that the movement of HMBS domains 1 and 2 relative to domain 3 is constrained by an “insertion region” (residues 296–324), which is absent in bacterial HMBS orthologs.^49^ We performed MD simulations (see material and methods) that confirmed both this and the previous suggestion that accommodation of the elongating polypyrrole is assisted by movement of the HMBS cofactor-binding loop in concert with the active-site loop and insertion region (Figure S5).^33^^,^^61^ Our maps find the insertion region to be generally tolerant of variation (Figure S6), consistent with a role for the insertion region as a volume-filling “wedge” that separates domain 3 from domains 1 and 2, allowing room for the elongating polypyrrole (a role that does not depend strongly on the precise biochemical nature of specific insertion region residues). Interestingly, a strong functional impact was observed for a set of mutations at the interface of domain 1 and 3: Thr109, Ile110, and Ile113 in domain 1 and Gly317, Ile318, Thr319, and Ala320 in domain 3 (Figure S6), suggesting that coupling of the mobility of domains 1 and 3 may be more important than previously appreciated.

Mutating the above-mentioned glycine in hinge 3-3 to a proline (p.Gly346Pro) can potentially restrict the flexibility of the backbone and consequently can affect the enzymatic function. The hydrogen bonding pattern of residues at the C-terminal 3-3 hinge region was considerably impacted by the introduction of p.Gly346Pro mutation (Table S4). Our MD simulations showed more persistent interactions between Arg355 and Leu257, Gly259, and Asp352 (Table S5) and correspondingly reduced flexibility along the C-terminal helix (Figure S7; Table S5). Increased rigidity of the C-terminal helix inhibited mobility of the cofactor-binding loop, which presumably affects accommodation and stabilization of the substrate. Indeed, the Asp99-substrate interaction was significantly reduced in the p.Gly346Pro variant relative to WT (Table S3)**.**

### Further interrogating key active site loop residues via simulated molecular dynamics

The active site loop, in addition to its roles in recruitment of PBG and chain elongation, has been implicated in two of the three pathways proposed for HMB’s exit from the active site.^49^ Here, we used MD simulation to explore two additional hypotheses related to the active site loop.

First, on the basis of analysis of the known structure, we hypothesized that a salt bridge between Asp61 and Lys27 controls flexibility and the positioning of the active site loop. Three HMBS variants—p.Asp61Asn, p.Asp61Ala, and the double mutant p.Asp61Lys;Lys27Asp (representing a “swap” of the amino acids at these two positions) —were investigated virtually with a 500-ns MD simulation. To quantify the relationship between the formation of the Asp61-Lys27 salt bridge and the position of the active site loop, the distances between residue pairs Gly60-Arg26 and Gly60-Gln34 were measured, and we considered the Asp61-Lys27 salt bridge to be present (i.e., in the “closed” conformation) if we observed an Asp61-Lys27 distance was equal or below 4 Å and absent (the “open” conformation) if above 4 Å.

Our simulation showed that the WT apoenzyme tends to maintain the Asp61-Lys27 salt bridge, with a mean distance of 3.5 ± 1.8 Å between Asp61 and Lys27 residues, spending ∼70% of simulation time in the closed state (Figures S8 and 3). Simulations of the p.Asp61Asn, p.Asp61Ala, and p.Asp61Lys;p.Lys27Asp variant structures showed that, for each variant, the distance between salt bridge residues increased (10.7 ± 2.2, 8.4 ± 1.6 Å, and 5.4 ± 2.4 Å, respectively; Figure S8). The average Gly60-Arg26 and Gly60-Gln34 distances also increased (Table S6), with the active site loop of the single mutants remaining in the “open” state (both for ∼100% of simulation time), while the double mutant (p.Asp61Lys;Lys27Asp) was in the closed state ∼30% of the time. Because the p.Asp61Lys-p.Lys27Asp amino acid swap by itself should not have significantly affected salt bridge formation, the observation that the swap induces the open state suggests other roles for at least one of these residues. One possibility is that Asp61 has an alternative salt bridge partner, Arg26 (adjacent to Lys27), with hydrogen bonding to the backbone of position 27. This idea that an Asp61-Arg26 salt bridge can substitute for that of Asp61-Lys27 is supported by our observation that, while Lys27 is quite tolerant, Asp61 is generally sensitive to variation. Although the average distance between Asp61 and Arg26 for the WT protein is ∼4 Å greater than that of Asp61 and Lys27 (3.5 Å), the electrostatic interaction between Asp61 and Arg26 side chains is noticeable. To further explore this hypothesis, we simulated the effects of a p.Arg26Pro variant that should disrupt a Asp61-Arg26 salt bridge and found that it greatly increased the average Asp61-Lys27 distance in the p.Arg26Pro variant (10.2Å ± 3.1 Å) compared to that of WT (3.5 ± 1.8 Å). An Asp61 role in active site loop conformation may explain its sensitivity to variation, while the presence of Arg26 can mitigate the impact of variation at Lys27 by providing an alternative salt bridge partner for Asp61.

**Figure 3:**
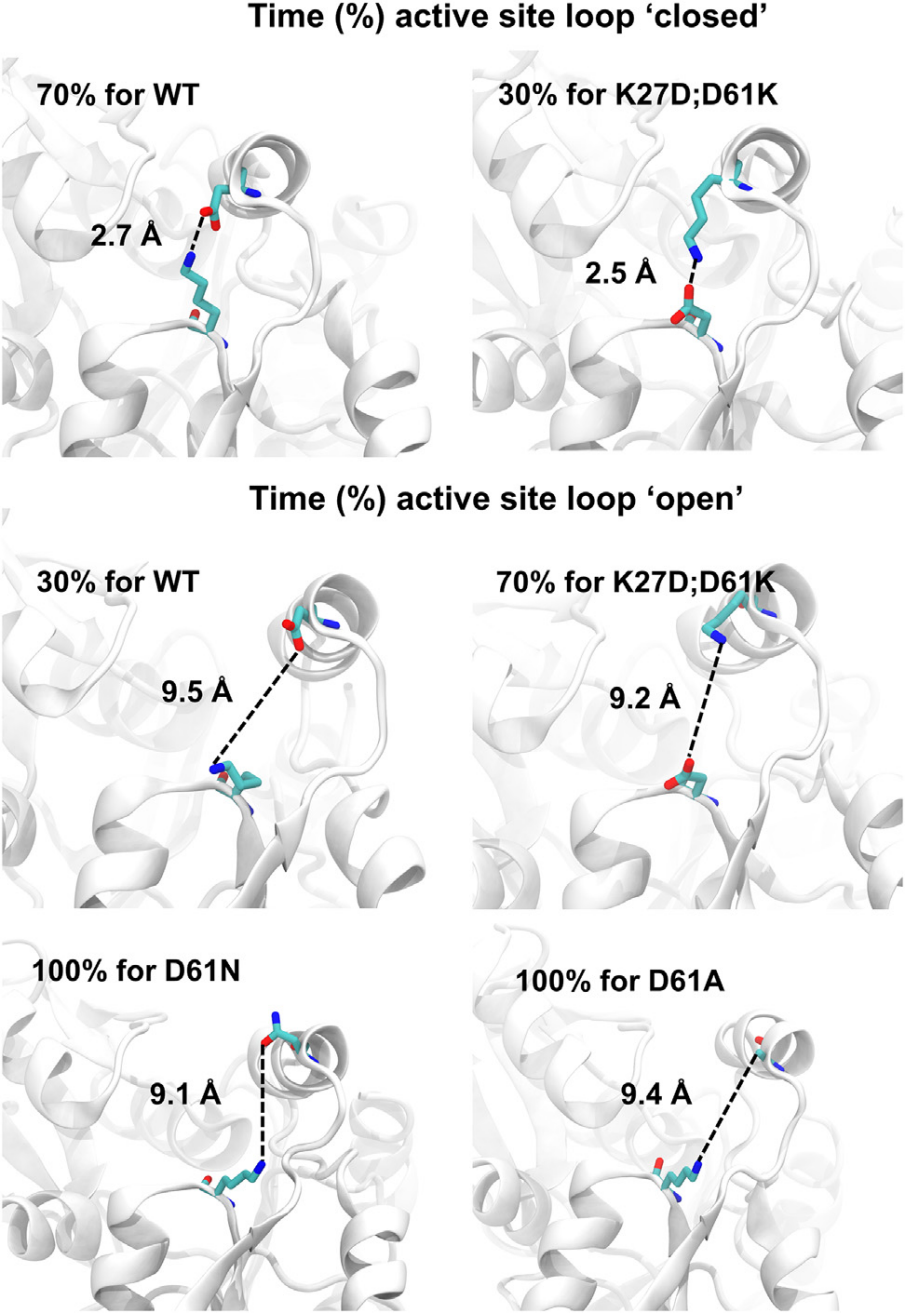
Variant impact on “closed” and “open” active site loop conformations The average distance (Å) between protein position 27 and 61 is shown, along with fraction of time spent in each conformation, for WT HMBS and three HMBS variants—p.Asp61Asn, p.Asp61Ala, and the double mutant p.Asp61Lys;Lys27Asp.

Our second hypothesis was that active site loop residue Gly60, which was striking in its intolerance to mutation, is important due to the backbone flexibility it provides. To explore this hypothesis, we simulated the impact of a p.Gly60Pro substitution that would be expected to constrain backbone motion. These simulations showed that p.Gly60Pro inhibits the Asp61-Lys27 salt bridge, likely via the induced rigidity of the loop that hinders the orientation of Asp61 needed for the Lys27 salt bridge (Figure S9). Furthermore, an Asp65-Lys27 salt bridge was observed for 91% of simulation time for the p.Gly60Pro mutant (Figure S8), which serves to constrain the active site loop to the “open” state (Table S6).

Taken together, the MD simulation results suggest that Lys27, Gly60, and Asp61 each play an important role in the flexibility of the active site loop, thereby impacting the uptake of PBG subunits and/or exit of the processed substrate. Distances between Asp61 and other residues observed in simulation are summarized in Table S6.

### Comparing measured functional impacts with predicted protein stability effects

**Figure 4:**
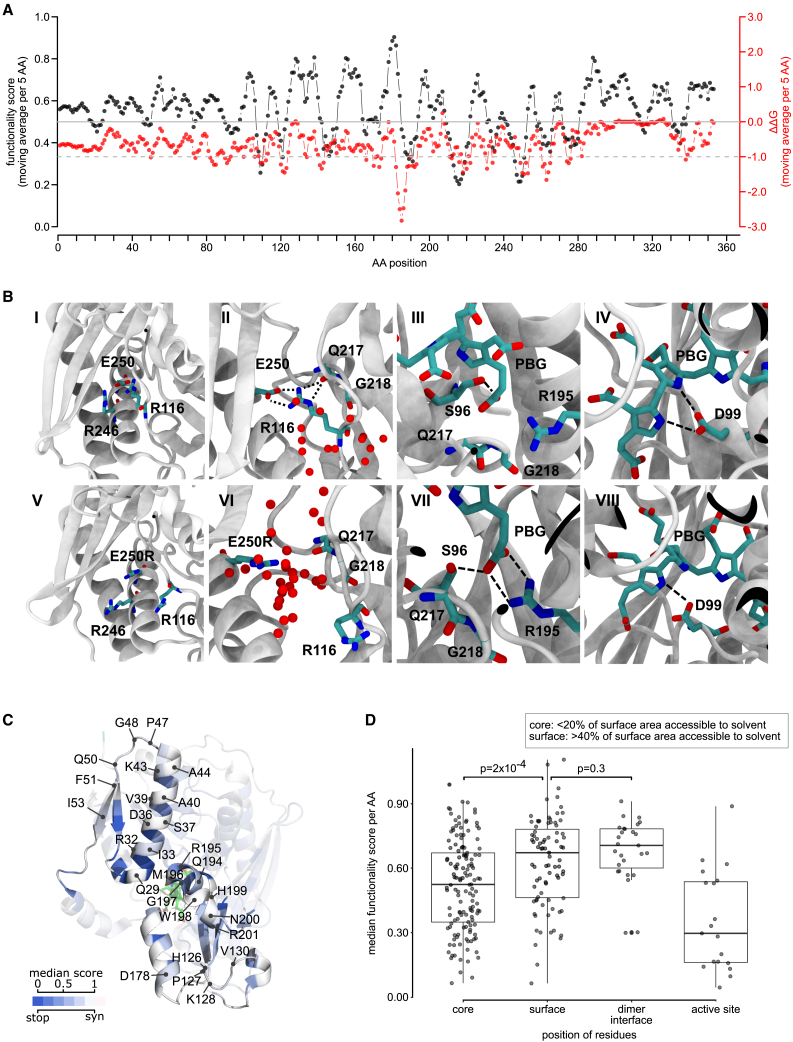
Modeling the effects of HMBS missense variants on protein stability and structure (A) Comparison of functional impact scores (black) and predicted free energy change (ΔΔG; red) values of HMBS missense variants. Plotted values are averages within windows of five amino acid (AA) positions. (B) WT (top; I–IV) and p.Glu250Arg variant (bottom; V–VIII) comparison. The p.Glu250Arg substitution repels Arg116 and opens a channel (V), which is exposed to solvent (VI). The PBG-Gly218 interaction is lost in the p.Glu250Arg variant (VII) and replaced by a salt bridge between PBG and Arg195, which in turn disrupts Asp99-pyrrol interactions (VIII). For clarity, hydrogen atoms are not shown. Water molecules (represented as red spheres) that are within 7 Å of either Glu250/Arg250 or Gln217 for more than 50% of the simulation time are shown. (C) Structural model of HMBS; colored according to the median functionality score of substitutions at each position, along with a wireframe model of the tetrapyrrole (green), and noting residues located at the dimer interface. (D) Median functionality scores of variants at amino acid positions that were (1) below 20% accessible surface area (ASA), (2) above 40% ASA, (3) at the dimerization interface with a threshold ΔASA of 1.0, and (4) active site residues required for polypyrrole assembly. Boxes correspond to interquartile range, and bold bars indicate medians. Whiskers correspond to minima and maxima. p values were calculated by Mann-Whitney U test.

It can be instructive to compare variant impacts on stability (ΔΔG) as opposed to overall functionality. For example, it has been shown that mutations having an impact on function but not on stability are more likely to be active site residues.^64^^,^^65^ We therefore performed moving window analyses enabling comparison of map scores and predicted ΔΔG values at different protein positions. As expected, these profiles were correlated with one another. Protein positions with predicted-destabilizing substitutions tended to have deleterious functional impact scores (Figure 4A). Surface residues (here defined by solvent-accessible surface area [ASA] < 20%) tended to have neither predicted stability effects nor strong functional impacts. In contrast, residues with high functional impact that were not predicted to have strong stability effects were restricted to active site residues involved in polypyrrole elongation (Arg26, Thr145, Ser147, Arg149, and Arg150) and to positions 316–319 (Figure S4). Positions 316–319, located outside the active site at the interface of domain 3 and domain 1, are packed closely and exhibit low mobility in our MD simulations (Figure S5). An impact of changes in residues 316–319 to function but not stability, coupled with their involvement in inter-domain residue-residue interactions (including Thr109-Thr319, Ile113-Ile318, and Ile110-Ile318), suggests that they help restrain structural fluctuations that would otherwise reduce enzymatic activity.

To further explore the importance of inter-domain residue interactions on structural dynamics, we used MD simulations to examine the variant p.Thr319Gln, which our map found to be damaging. Root-mean-square fluctuations of the Cα atoms of all amino acids throughout the simulation time (RMSF) were examined, both in the context of the WT and p.Thr319Gln structures. As expected, RMSF values tended to be higher for p.Thr319Gln in all three domains, with a significant increase for residues in the active-site loop and the insertion regions (Figure S6). Interestingly, the p.Thr319Gln structure exhibited lower RMSF values within the cofactor-binding loop. Because the cofactor-binding loop (residues in positions 257–263) normally moves during catalysis to accommodate shifting of the initial DPM substrate as the polypyrrole elongates,^49^^,^^61^ we conclude that the p.Thr319Gln variant hinders cofactor-binding loop movement that would otherwise accommodate polypyrrole chain elongation.

We next assessed whether salt bridges outside the active site may be important for protein folding and/or stabilization. Mutations predicted to disrupt the formation of each of several salt bridges—Arg116-Glu250, Asp121-Arg149, Asp121-Arg150, and Arg225-Asp91—appeared damaging in our maps (Figure S6). Because both the Arg116 and Glu250 positions are associated with pathogenic variation (p.Arg116Trp, p.Arg116Gln, and p.Glu250Lys), we focused our attention on the corresponding Arg116-Glu250 salt bridge. In addition to this salt bridge, hydrogen-bonding of Arg116 to the Gln217 backbone keeps Gln217 in place and aligns the Gly218 side chain to effectively interact with a PBG carbonyl group (Table S3; Figure 4B). To further evaluate the importance of the Arg116-Glu250 salt bridge, we performed MD simulations for variant p.Glu250Arg. Here, we observed the p.Glu250Arg mutation to eliminate the Glu250-Arg116 salt bridge, with the Arg250 mutant residue opening a water-filled channel between the helix (224–240) and the loop (198–202) (Figure 4B) and repelling Arg116. In the p.Glu250Arg simulation, we also observed water molecules hydrogen bonded to both Arg116 and Gln217, thus replacing the Arg116-Gln217 interaction and eliminating the Gly218-PBG interaction. We attribute this effect to increased active site loop flexibility caused by p.Glu250Arg (Figure 4B). The simulation also suggested that loss of the Glu250-Arg116 salt bridge drives a new salt bridge between PBG and Arg195, in turn altering the position of PBG and weakening hydrogen bonding between Asp99 and pyrrole groups (Figure 4B). Thus, the combination of our map and simulation analysis suggests that p.Glu250Arg not only disrupts Arg116-Glu250 salt bridge and other local structure around position 250 but also causes a cascade of changes in backbone and side chain conformations, altering residue interactions in the active site to impact both protein stability and position of the tetrapyrrole substrate.

### Evaluating HMBS missense impacts in the context of the homodimeric HMBS structure

Examining whether protein core residues are more sensitive to mutation than surface residues, we found buried residues (those with <20% ASA) to have a lower median score than surface residues (>40% ASA; Δmedian = 0.13; p = 0.0002 Mann-Whitney U test; Figure 4D). Interestingly, interface residues, defined as those with |ΔASA| > 1 Å2, exhibited scores that were similar to other surface residues (Δmedian = 0.04; p = 0.3; Mann-Whitney U test; Figure 4D).

Although HMBS has been reported to function as two monomers in an asymmetric unit with a weak dimer interface,^62^^,^^66^ the relationship between dimerization and the monomeric enzyme’s stability and activity remains unclear.^48^ To visualize missense functional impact scores in the context of the homodimeric HMBS structure, we colored each residue in the structure according to the median fitness of substitutions at that residue. Residues at the dimer interface—especially those at the center (Trp198 and His199)—appeared highly tolerant to variation (Figure 4C). One exception was residue Phe77 for which all variants scored as damaging. The sensitivity of Phe77 to variation (Figure 2B, V) can be explained by the interaction between residues Phe77 and Arg26, which helps maintain the active-site loop in a closed conformation through the first stage of chain elongation (Figure S9B).^49^^,^^67^ In fact, our MD simulation data indicate that eliminating the cation-π interaction in the p.Phe77Ala variant increased the Asp61-Arg26 and Asp61-Lys27 distances (both by ∼ 3 Å) and exposed the active site, confirming the importance of Arg26-Phe77 interaction. Thus, Arg26 is not only involved in the salt bridge mentioned above, but also provides structural integrity via interaction with Phe77. Given that the only residue at the dimer interface exhibiting substantial sensitivity to variation can be explained without dimerization, our results support a previous hypothesis that HMBS dimerization is not critical for its stability.^48^

### Limited correlation between functional impact scores and disease severity

For genetic diseases broadly, penetrance and expressivity can depend on the extent to which a variant impacts the function of the associated gene^26^ or have other explanations (e.g., additional genetic variation or environmental causes). For AIP, the low penetrance and variable expressivity of AIP even amongst individuals harboring a shared HMBS missense variant (e.g., p.Arg167, p.Arg173, p.Arg225, and p.Arg325)^3^ suggests that extragenic variation and environmental differences are a more likely explanation. Nonetheless, we sought to examine the correlation between our functional impact scores and the age of onset or severity of AIP. Although there is no accepted framework for classifying AIP severity, one study has categorized individuals according to AIP severity, reporting that phenotypic severity correlated with a variant’s position relative to the active site.^68^ We adopted these assignments for our analysis of AIP severity but are compelled to note that no objective criteria for these assignments were described. The observation that our functional impact scores correlated poorly with age of disease onset and also with previously classified disease severity (Figures S11C and S11D) argues against strong dependence of AIP severity on quantitative differences in variant functional impact.

### Comparing measured functional impacts with population genotypes

While AIP exhibits incomplete penetrance, HMBS intolerance to variation is supported by a gnomAD “loss-of-function” intolerance (pLI) score > 0.95, a score that includes only nonsense and frameshift variants amongst loss-of-function variants. Similarly, missense variants called as damaging in our combined map were depleted in human cohort databases (see material and methods; OR = 3.34, p = 5 × 10^−11^; Fisher’s exact test; Figure S12B), suggesting that reduced-function HMBS variants have been counter-selected in the human population.

### HMBS functional impact scores identify pathogenic variants

**Figure 5:**
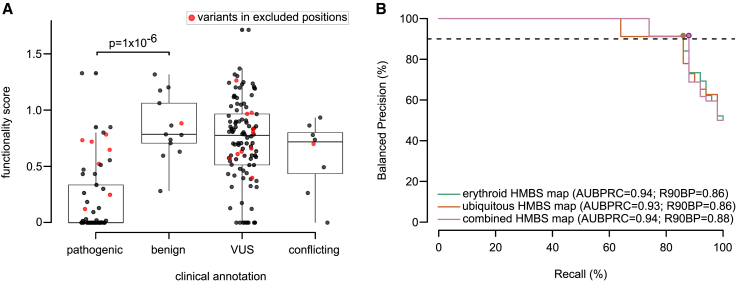
Performance of variant effect maps in distinguishing pathogenic from benign reference variants (A) The distribution of functional impact scores for reference “pathogenic,” “benign,” “VUS,” and “conflicting” variant sets. Variants from residue positions 160 to 215, 255, or 355 (indicated in red) were excluded from performance analysis given the suspected limitation of our assay for these variants. Boxes correspond to interquartile range, and bold bars indicate medians. Whiskers correspond to minima and maxima. Significance was evaluated with a Mann-Whitney U test. (B) Evaluation of precision (fraction of variants scoring below each threshold functionality score that are in the positive reference set containing pathogenic variants) vs. recall (fraction of positive reference variants with functionality scores below threshold). Here, precision values have been “balanced” to reflect performance in a setting where positive and negative sets contain the same number of variants. Balanced precision-recall curves are shown for erythroid-specific (green), ubiquitous (orange), and combined maps (pink). Performance is also described in terms of area under the balanced precision vs. recall curve (AUBPRC) and recall at a balanced precision of 90% (R90BP).

Beyond understanding sequence-structure-function relationships, variant effect maps offer functional evidence in support of clinical variant interpretation. To relate our HMBS maps with pathogenicity, we first assembled a positive reference set of 53 likely pathogenic or pathogenic missense variants and a negative reference set of 13 missense variants that were benign, likely benign, or “proxy benign” (see material and methods). Scores tended, as expected, to be lower for positive than negative reference sets (Δmedian = 0.7; p = 1 × 10^−6^; Mann-Whitney U test; Figure 5A). We then judged performance by using both receiver operating characteristic (ROC) analysis and precision (fraction of variants below a given threshold score that have been annotated as either pathogenic or likely pathogenic) versus recall (fraction of all variants annotated as pathogenic or likely pathogenic that scored below the threshold) analysis. Because precision depends on the proportions of pathogenic and benign variants in the reference set (which may not accurately reflect the prior probability that any given clinical variant is pathogenic or benign), we transformed each empirical precision vs. recall curve to the corresponding “balanced precision” vs. recall curve reflecting the precision that would be achieved with a prior that is balanced (i.e., where the prior probability of pathogenicity is 50% ; Figure 5B). We also measured the recall at a stringent balanced precision threshold of 90% (R90BP).^69^ Thus, our performance measures were as follows: area under the ROC curve (AUROC), area under the balanced precision recall curve (AUBPRC), and recall at 90% balanced precision (R90B). First, we observed statistically indistinguishable AUROC (p > 0.3; DeLong’s test; Figure S13) and AUBPRC (empirical p > 0.12; Figure S14) performances for all pairwise comparisons of our maps (Figure S13). Both erythroid-specific and ubiquitous maps captured 86% of scored pathogenic variants at high stringency (i.e., R90BP was 86%), while the combined map had an R90BP of 88% (Figure 5B). Interestingly, functional impact scores of annotated pathogenic and benign variants were generally well separated from one another except one region (residues 160–215) where known pathogenic variants exhibited limited functional impact (Figure S15). Agreement with the map seen for individual yeast complementation assays of variants in this region suggest that the map accurately reflects the yeast-based assay and is consistent with the hypothesis discussed above—that growth of the polypyrrole chain beyond tetrapyrrole may not be required to rescue the *HEM3-ts* phenotype (Figure S1). In summary, our HMBS variant effect map corresponds well with pathogenicity, with the important caveat that the results should not be taken to infer benignity for residues in positions 160 to 215, 255, or 355. After excluding these positions, the combined map had an R90BP performance of 93% (Figure S14).

Experimental and computational sources of evidence about variant pathogenicity are complementary, in the sense that current ACMG/AMP guidelines for variant interpretation consider these evidence types to be independent of one another. Previous observations found experimental functional assays to offer higher sensitivity than most computational predictors (e.g., PROVEAN, PolyPhen2, and SIFT)^24^^,^^25^^,^^26^^,^^28^^,^^70^^,^^71^^,^^72^ Considering all residue positions, our combined map was outperformed according to all criteria (i.e., AUBPRC, R90BP, and AUROC) by state-of-the-art computational predictors DeMaSk,^73^ ESM-1b,^52^ and VARITY_R.^69^ We evaluated the extent to which computational predictors’ success depended on the inclusion of positions 160 to 215 and positions 255 and 355. While sensitivity values with (and without) the inclusion of these positions—R90BP of 87% (93%)—were somewhat different for our map, this mattered less for computational predictions, with R90BP values of 96% (95%), 98% (98%), and 96% (95%) for DeMaSk, ESM-1b, and VARITY_R, respectively (Figure S14).

Finally, to enhance the accuracy and sensitivity of HMBS missense variant interpretation, we re-stated each variant’s fitness score in terms of a likelihood ratio of pathogenicity (LLRp). The LLRp value estimates the likelihood of obtaining the observed score in the positive reference set relative to the corresponding likelihood in the negative reference set, which can also be translated into discrete evidence strengths in the ACMG/AMP framework.^32^ This analysis suggests that LLRp scores above 0.64 should be treated as moderate evidence of pathogenicity, LLRp scores between 0.64 and 0.32 as supporting evidence of pathogenicity, and LLRp scores below −0.32 as supporting evidence of benignity (Figure S16).

Based on this calibration of LLRp scores to ACMG/AMP evidence strength categories, our map provides functional evidence for variant classification for 94 (82%) of the 115 HMBS missense VUSs reported in ClinVar. Of these 94 variants, 80 (85%) and 1 (1%) received scores corresponding to supporting or strong evidence of benignity (Table S8), while 3 (3%) and 10 (11%) received LLRp scores corresponding to moderate or strong evidence of pathogenicity, respectively.

## Discussion

By combining codon-randomizing mutagenesis and large-scale multiplexed functional assays, we proactively assessed the functional impact of missense variants in human *HMBS*, covering a large fraction of missense variants in both the erythroid-specific and ubiquitous HMBS isoforms.

Our functional impact scores agreed closely with known sequence-structure-function relationships, with some exceptions within the active site and active site loop and at the hinges controlling flexibility and dynamics of HMBS domains. For example, at positions important for DPM formation, binding, or chain elongation, we found missense variation to be damaging as expected. However, the maps showed some variants at positions involved in chain elongation (e.g., residues Ser165, Asn169, Arg255, and Arg355) to be surprisingly tolerated. These were generally at positions important for stabilizing the negatively charged polypyrrole, which is consistent with previous reports that water molecules can compensate for loss of charge stabilization.^49^ Some variants near the active site or within the active-site loop were also tolerated, and our results suggest that a critical determinant of mutational tolerance at these positions is the relative distance from the active site.

Based on our map scores and MD simulations, residue interaction at the interface of domain 1 and 3 modulates the dynamic behavior of HMBS during catalysis. Furthermore, our results demonstrate that a salt bridge network (Arg26, Asp61, Thr58, and Lys27) and cation-π interaction between Phe77 (part of the active-site loop) and Arg26 ensures the active-site loop remains in a “closed” conformation during cofactor assembly and the first addition of PBG.^49^ Further studies to confirm the validity of our MD simulation results might focus on investigating the role of the Asp61-Lys27 salt bridge in controlling the flexibility and positioning of the active-site loop, e.g., our results would predict *in vitro* enzymatic studies of the amino-acid-swapping double-mutant p.Asp61Lys;Lys27Asp to show minimal impact on protein activity or thermostability. However, the electrostatic interaction between the Asp61 and Arg26 side chains might alternatively be crucial within a complex salt bridge, leading to inactivity for the p.Asp61Lys;Lys27Asp double mutant. Furthermore, when examining the general contribution of salt bridges (Glu250-Arg116, Asp121-Arg150, Arg225-Asp91, and Arg149-Asp121) toward overall protein stability, the maps showed variation at such positions to be damaging, resulting in large-scale structural rearrangements in the backbone conformation and side chains’ geometrical orientation that would impair catalytic activity.

The HMBS variant effect maps revealed other patterns of mutational tolerance. For example, variants at the dimerization interface were generally tolerated, suggesting HMBS does not require dimerization for its function. Moreover, variants at non-dimerization-interface surface residues tended to be strongly damaging, perhaps due to introduction of hydrophobic residues that favor aberrant folding. Both of these findings are consistent with suggestions by Chen and colleagues based on functional assays of 11 missense variants.^48^

One caveat of our map is that our functional complementation assays are based on the expression of mature cDNA, so that any impact of variants on splicing will necessarily have been missed. Because introns are important for nonsense-mediated decay in mammalian cells, it is possible that nonsense variants seen as tolerated in a yeast-expressed cDNA would be damaging in the endogenous human context. Nevertheless, nearly all nonsense variants were found in our yeast cDNA assay to be highly damaging (Figure 1C).

An important limitation of our maps is that it measures total activity, not specific activity. Thus, we cannot distinguish functional impacts on protein abundance, e.g., due to misfolding that accelerates protein degradation, from impacts on specific activity that do not affect abundance. Our observation that the correlation between functional impact scores and *in vitro* measurements of specific activity was significant but moderate (Spearman’s ϱ *=* 0.54, p = 4 × 10^−9^, Figure S11A), together with a similar observation for correlation of functional impact scores with predicted impacts on thermodynamic stability (Spearman’s ϱ *=* 0.52, p = 2 × 10^−16^, Figure S11B), suggests that both stability and specific activity effects are at play. For the purpose of understanding sequence-structure-function relationships, it would be interesting to determine whether impacts on total activity arose via impact on steady-state protein level (as could be measured for example by the VAMP-Seq method^22^) or via impact on specific activity. However, it is not clear that knowing whether total HMBS activity is lost due to reduced expression levels as opposed to specific activity would have clinical value.

Another important caveat is that our measurements were necessarily subject to both systematic and random error. We used previously described methods^28^ to estimate random error for each experimental score, reflecting the estimated magnitude of random experimental error. Systematic errors could have arisen from many sources. For example, any errors made in recalibrating the score range for each region (see material and methods) could cause scores from one region to be systematically higher or lower than another. Some systematic errors may also have arisen from the nature of our assay. For example, we observed some residues implicated in the addition of the final two PBG monomers to be surprisingly well tolerated in our yeast-based assay, which could be explained if residual activity of the yeast *HEM3* ts mutant can extend (but not generate) HMB. Because many substitutions in 3D proximity to residues known to be important for stabilization of the polypyrrole chain (e.g., Arg167, Ser165, Asn169, Arg255, and Arg355) were found tolerated by our assay, we suggest that positions 160–215, 255, and 355 in the map be excluded when inferring benignity. However, this map region could remain useful for inferring pathogenicity.

Despite these limitations, we found that our variant effect maps could reliably discriminate pathogenic or likely pathogenic from benign, likely benign, or “proxy benign” variants, and the best performance came from the combined map, surpassing that of HMBS activity assays (empirical p = 0.043; Figure S14) as well. When comparing our maps with computational predictors at thresholds providing equally stringent (90%) precision, our maps captured a slightly lower number of pathogenic variants compared to three examples of the most up-to-date computational predictors: DeMaSk, ESM-1b, and VARITY. Although the performance of each map was improved further by excluding residues 160–215, 255, and 355, the overall conclusions in the comparison with computational methods did not change. This comparison should not be taken to suggest that there must be a competition between computational and experimental evidence. Indeed, treating these evidence sources independently per ACMG/AMP guidelines means that these evidence sources can be synergistic, albeit with a currently greater evidence strength afforded to experimental assays of variant function.^15^

An important caveat of variant interpretation is that variants determined to be pathogenic (whether via variant effect maps or otherwise) may not cause disease in every individual. This caveat is particularly pronounced for pathogenic HMBS variants, which generally have low penetrance. Our study was also limited by the number of publicly available rare missense variants that have been annotated as “likely benign” or “benign” in ClinVar. We used HMBS variants in gnomAD as a negative reference set after excluding those reported to be pathogenic or likely pathogenic. While we cannot exclude the possibility that some of these individuals have AIP (despite the rarity of this condition), variants in this negative reference set can at least be expected to be strongly depleted for pathogenicity.

Some variants in the C-terminal region of HMBS appeared to “hyper-complement,” i.e., grow faster than the WT control in the complementation assay, especially in the ubiquitous isoform map. Hyper-complementation could indicate increased activity in humans, e.g., possibly arising from increased stability or enhanced conformational flexibility and concomitant increase in catalytic activity. However, previous analyses of a missense variant effect map for the protein UBE2I (also based on a yeast assay) suggested both that *UBE2I*-hyper-complementing variants tend to be deleterious in humans and that they result from changes that are specifically adaptive in the yeast cellular context.^28^ Both phylogenetic analysis and a comparison with a high-performing computational predictor suggested that variants that are hyper-complementing in our assay, although we cannot be sure whether they tend to have gain- or loss-of-function impacts, are likely to be deleterious in humans.

Although we observed no significant correlation between functional impact scores and either age of disease onset or disease severity, we do not wish to suggest that this question is closed, absent more data and an accepted objective framework for classifying AIP severity. It would be interesting to investigate whether the functional impact scores are more predictive of age of onset or severity after stratifying by the presence or absence of known triggers.^1^

The HMBS variant effects maps we provide could have immediate value in several clinical scenarios. First and most importantly, where an individual has been diagnosed with AIP on the basis of clinical and biochemical data but has an *HMBS* missense variant that would otherwise be classified as a VUS, a more definitive classification of the variant could enable cascade screening to identify family members with latent AIP. Second, where an individual has highly elevated ALA and PBG but does not have access to a laboratory capable of ruling out the two other acute porphyrias, identification of a definitively classified HMBS variant can establish the correct diagnosis. Third, where an individual has a clinical history consistent with AIP but timely measurements of porphyrin precursors in urine or plasma were not obtained or were inconclusive. Fourth, where an *HMBS* missense variant is revealed, e.g., through direct-to-consumer genome sequencing, and the client wishes to know if they should be vigilant for symptoms of AIP or avoid its known triggers. In each of these scenarios, a resulting diagnosis of AIP or latent AIP could have the cascading benefit of helping to identify additional cases of AIP or latent AIP in at-risk relatives, thereby increasing the number of individuals for whom vigilance, prevention, or therapy is supported.

In addition to providing a resource for the understanding of HMBS variation, this study also provides proof of principle for broader application of variant effect mapping to other genes associated with acute hepatic porphyria.

In conclusion, we strengthen the evidence that systematic proactive experimental evaluation of missense variant effects on human enzymes can reveal sequence-structure-function relationships and yield clinically relevant insights with potential to guide personalized clinical decisions.

## Data Availability

Custom scripts for all downstream analyses are publicly available: https://github.com/wvanlogg/HMBS. Functional impact scores for the erythroid, ubiquitous, and combined variant effect maps have been deposited on MaveDB^54^ under accession number urn:mavedb:00000108-a. Genotypes and phenotypes for individuals with AIP drawn from Ipnet (https://porphyrianet.org/en/content/worldwide-network) and literature-curation, respectively, can be found in Table S7.

## Web resources

TileSeq_MutCount, https://github.com/RyogaLi/tileseq_mutcount
TileSeqMave, https://github.com/jweile/tileseqMave
Phydms, https://github.com/jbloomlab/phydms

## Supplemental information

Document S1. Figures S1–S17 and Tables S1–S6

Table S7. Curated reference set of disease- and non-disease-associated variants

Table S8. Log likelihood ratios of pathogenicity derived from the combine HMBS map

Table S9. List of POPCode mutagenesis oligos and TileSeq primers

Table S10. Ensembl homologs having at least 85% sequence identity to the human HMBS protein

Document S2. Article plus supplemental information
