# Supplementary material for: Systematically testing human HMBS missense variants to reveal mechanism and pathogenic variation
Source: Am J Hum Genet. 2023 Sep 19;110(10):1769–86. doi: 10.1016/j.ajhg.2023.08.012 (PMC10577081; doi:10.1016/j.ajhg.2023.08.012)
Supplement: Document S1. Figures S1–S17 and Tables S1–S6 [file mmc1.pdf]

The American Journal of Human Genetics, Volume *110*

Supplemental information

Systematically testing human HMBS missense variants to reveal mechanism and pathogenic variation

Warren van Loggerenberg, Shahin Sowlati-Hashjin, Jochen Weile, Rayna Hamilton, Aditya Chawla, Dayag Sheykhkarimli, Marinella Gebbia, Nishka Kishore, Laure Frésard, Sami Mustajoki, Elena Pischik, Elena Di Pierro, Michela Barbaro, Ylva Floderus, Caroline Schmitt, Laurent Gouya, Alexandre Colavin, Robert Nussbaum, Edith C.H. Friesema, Raili Kauppinen, Jordi To-Figueras, Aasne K. Aarsand, Robert J. Desnick, Michael Garton, and Frederick P. Roth

Supplemental figures

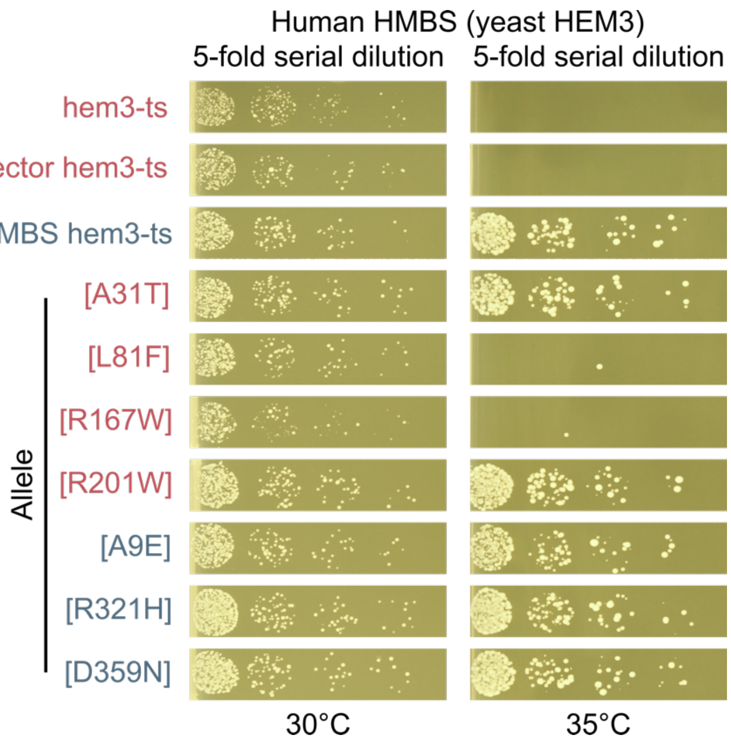

Figure S1. Functional complementation assay results showing whether expression of human HMBS protein variants can rescue growth of a yeast strain bearing a temperature-sensitive mutation in the essential gene *HEM3*. Pathogenic variants p.Ala31Thr, p.Leu81Phe, p.Arg167Trp and p.Arg201Trp and negative controls are indicated in red text, while benign variants p.Ala9Glu, p.Arg321His and p.Asp359Asn and a wild-type HMBS control are indicated in blue text. Fivefold serial dilutions of yeast cells were spotted onto plates, with growth evaluation after 48 hours of incubation at either permissive (30°C) or non-permissive (35°C) temperature.

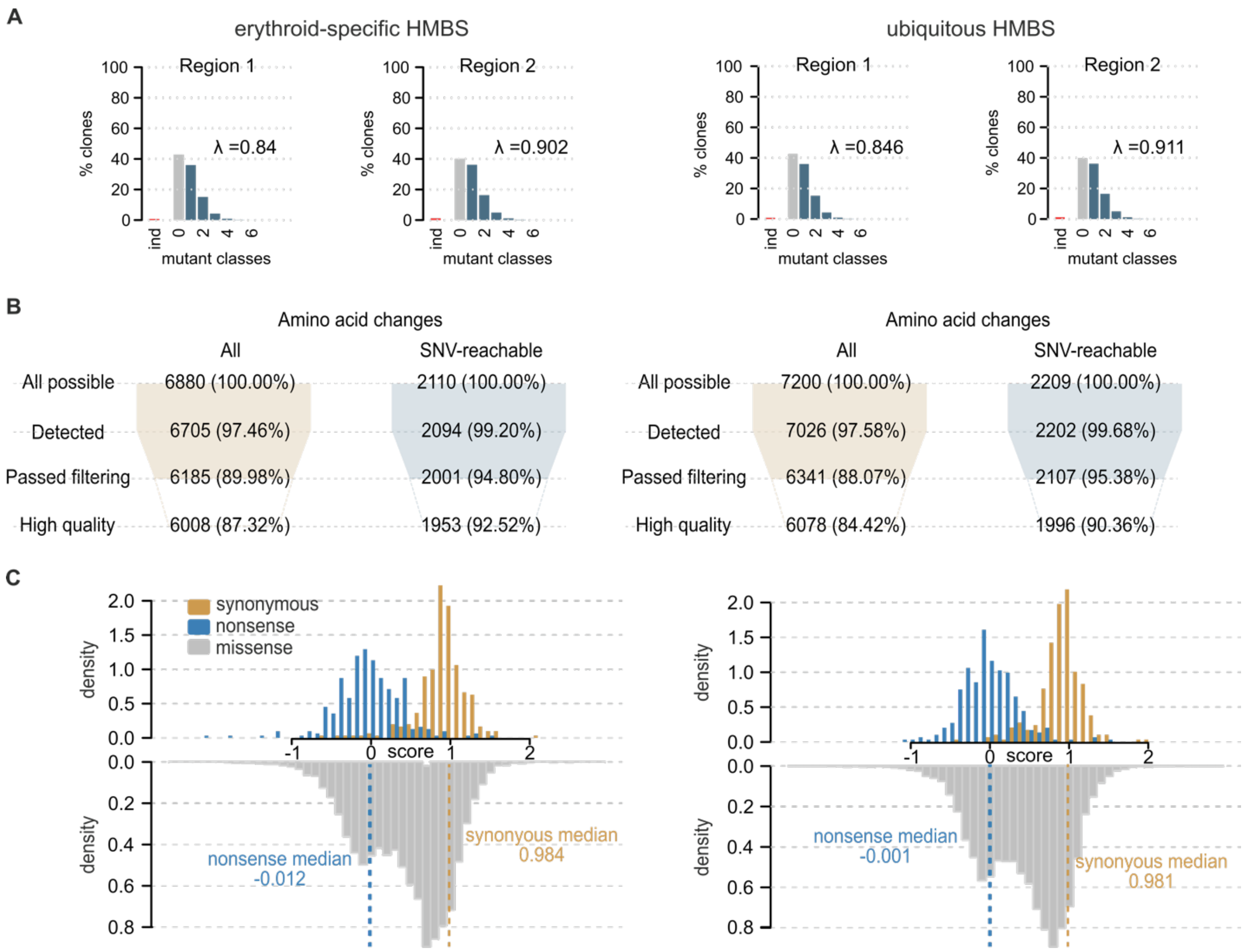

Figure S2. HMBS variant libraries underlying the combined variant effect map. (A) Distribution of the number of missense variants in clones from the erythroid-specific (left) and ubiquitous (right) HMBS mutagenized libraries, and the fraction of clones carrying small indels (“ind”). The average number of amino acid changes per clone (λ) is also estimated (see Methods). (B) Fractions of variants detected and passing after each of two levels of quality control are shown for each HMBS isoform. For each isoform, the number of synonymous, nonsense, and missense substitutions (up to 19 possible) are shown across all residue positions, both before (left) and after (right) restricting to substitutions that are possible given a single nucleotide change. The four rows correspond to: 1) theoretically possible substitutions; 2) substitutions detected in the non-selective condition; 3) substitutions above a threshold pre-selection frequency; and 4) substitutions with a sufficiently low regularized standard error. (C) Distributions of measured functional impact scores for nonsense, synonymous, and missense variants for the erythroid-specific (left) and ubiquitous (right) HMBS isoforms.

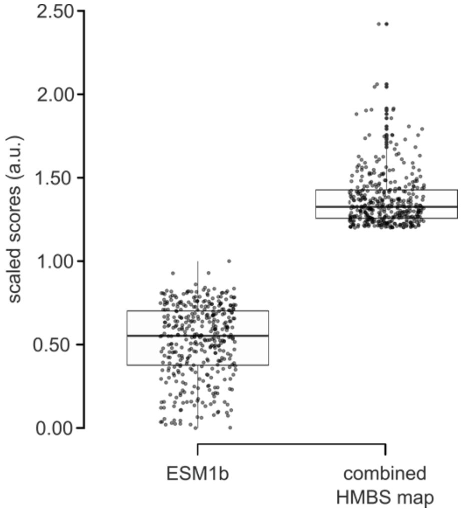

Figure S3. Comparison of 'hyper-complementing' scores from our combined map and the computational predictor ESM-1b (after transformation to a common scale). In this normalization, 0 represents null-like variants, scores near while 1 represents neutral variants, and scores significantly greater than 1 represent hyper-complementing behavior that our phylogenetic analysis suggests are deleterious in humans. Boxes indicate interquartile range, with bold horizontal lines indicating medians. Whiskers indicate maxima and minima inside 1.5x interquartile range.

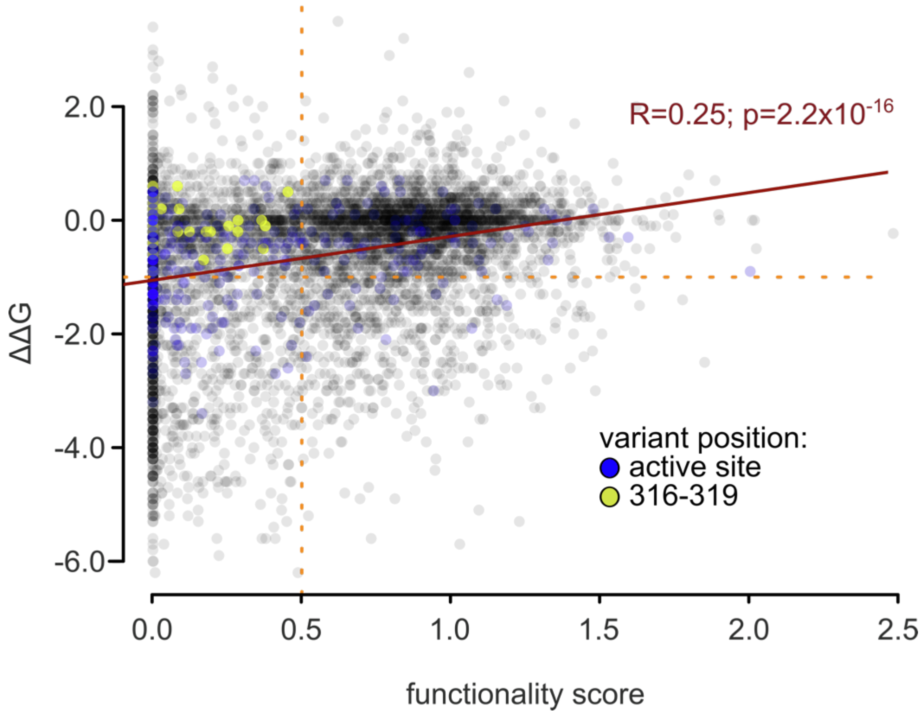

Figure S4. Correspondence between functional impact scores and predicted free energy change (ΔΔG) values of HMBS missense variants. For reference, variants at the active site (blue) and those in positions 316-319 that modulate HMBS structural fluctuations (yellow) are indicated, while the red line corresponds to a linear regression fit (R=0.25; P = 2.2×10^{-16}).

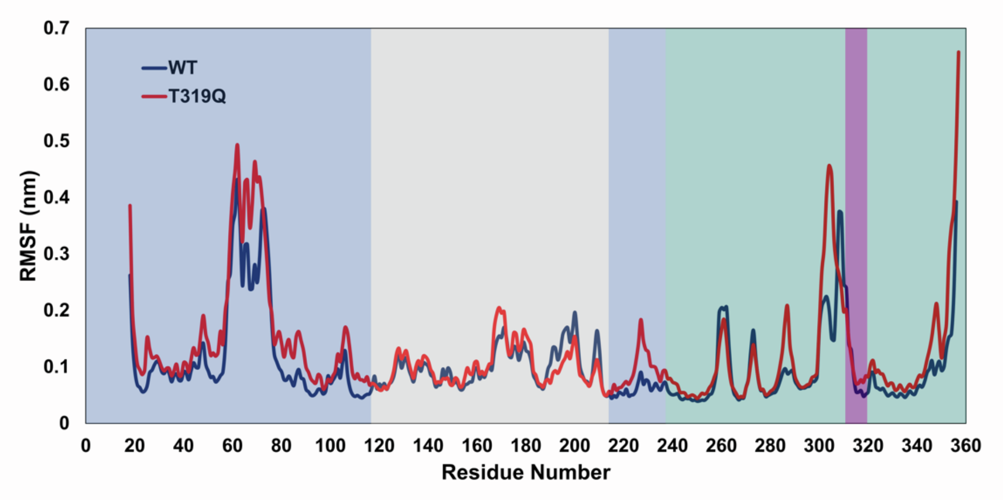

Figure S5. Root-mean-square fluctuation (RMSF) of Cα atoms of WT HMBS and the p.Thr319Gln variant. RMSF reveals the average deviation of atoms throughout the simulation with respect to the initial structure. Domains 1, 2, and 3 are highlighted in light blue, gray, and green, respectively. The 316-319 positions at the interface of domains 1 and 3 are highlighted in purple.

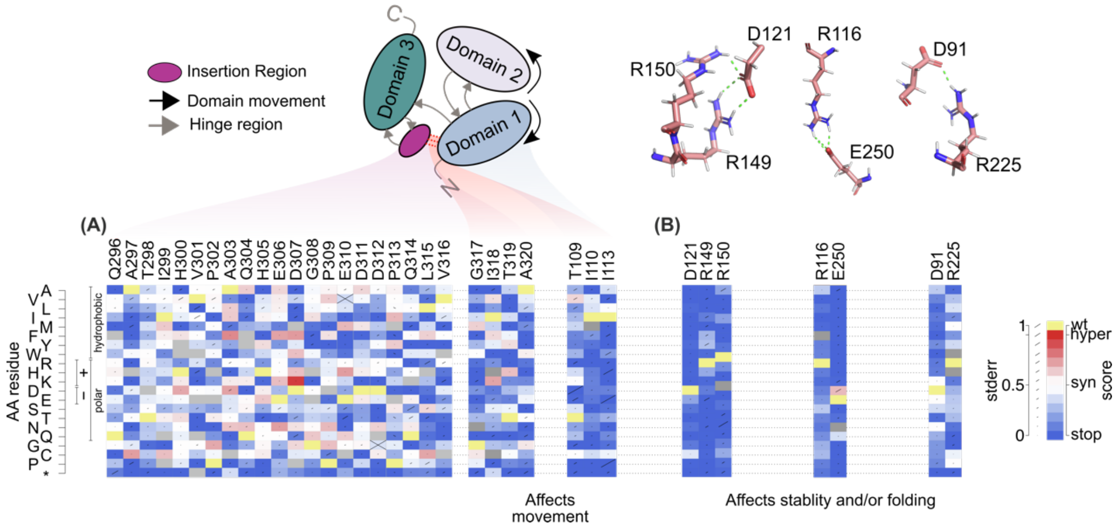

Figure S6. Placing functional impact scores in the context of residue roles in moderating HMBS fluctuation, protein stability or folding. Functional scores for each possible substituted amino acid (*y*-axis) at each residue position outside the active site (*x*-axis) participating in: (A) constraining HMBS domain movement, or (B) salt bridges. For each substitution, diagonal bar sizes convey estimated measurement error in the corresponding functional score. Box color either indicates the wild-type residue (yellow), or a substitution with damaging (blue), tolerated (white), or above-wildtype ('hyper-complementing', red) functional score, or missing data (gray).

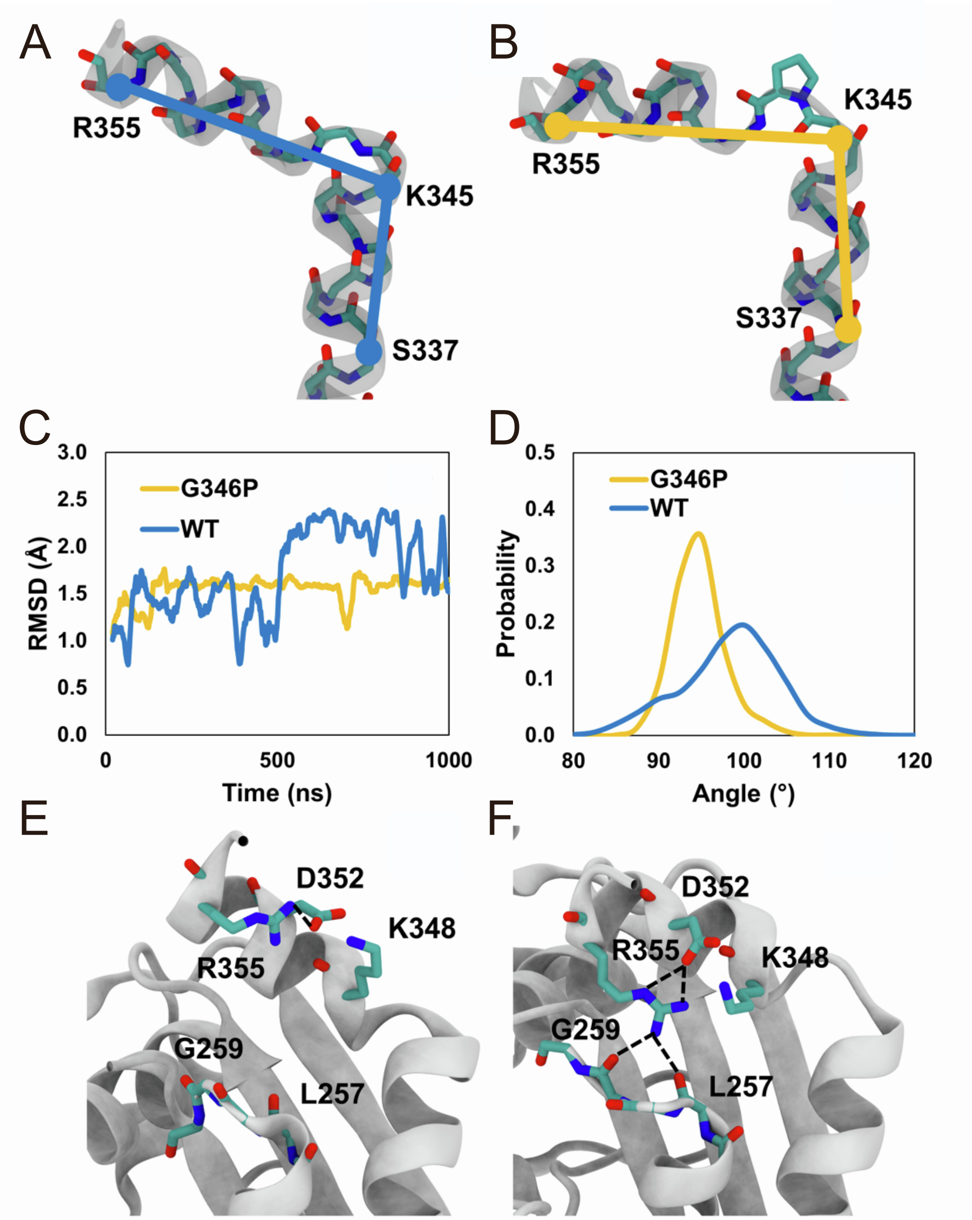

Figure S7. Comparison between WT and the p.Gly346Pro clinical variant showing a widespread impact on protein flexibility. Backbone atoms of Ser337, Lys345, and Arg355 were selected to evaluate the angle between the two helices in (A) WT and (B) p.Gly346Pro. For clarity only the C-terminal helices are shown. (C) The root-mean-squared deviation (RMSD) of the backbone atoms in the C-terminal helix (Lys345-Leu357). (D) Angle distribution for WT (average: 100° ± 6) and Gly346 (average: 96° ± 3). Interactions of Arg355 with neighboring residues in (E) WT and (F) p.Gly346Pro.

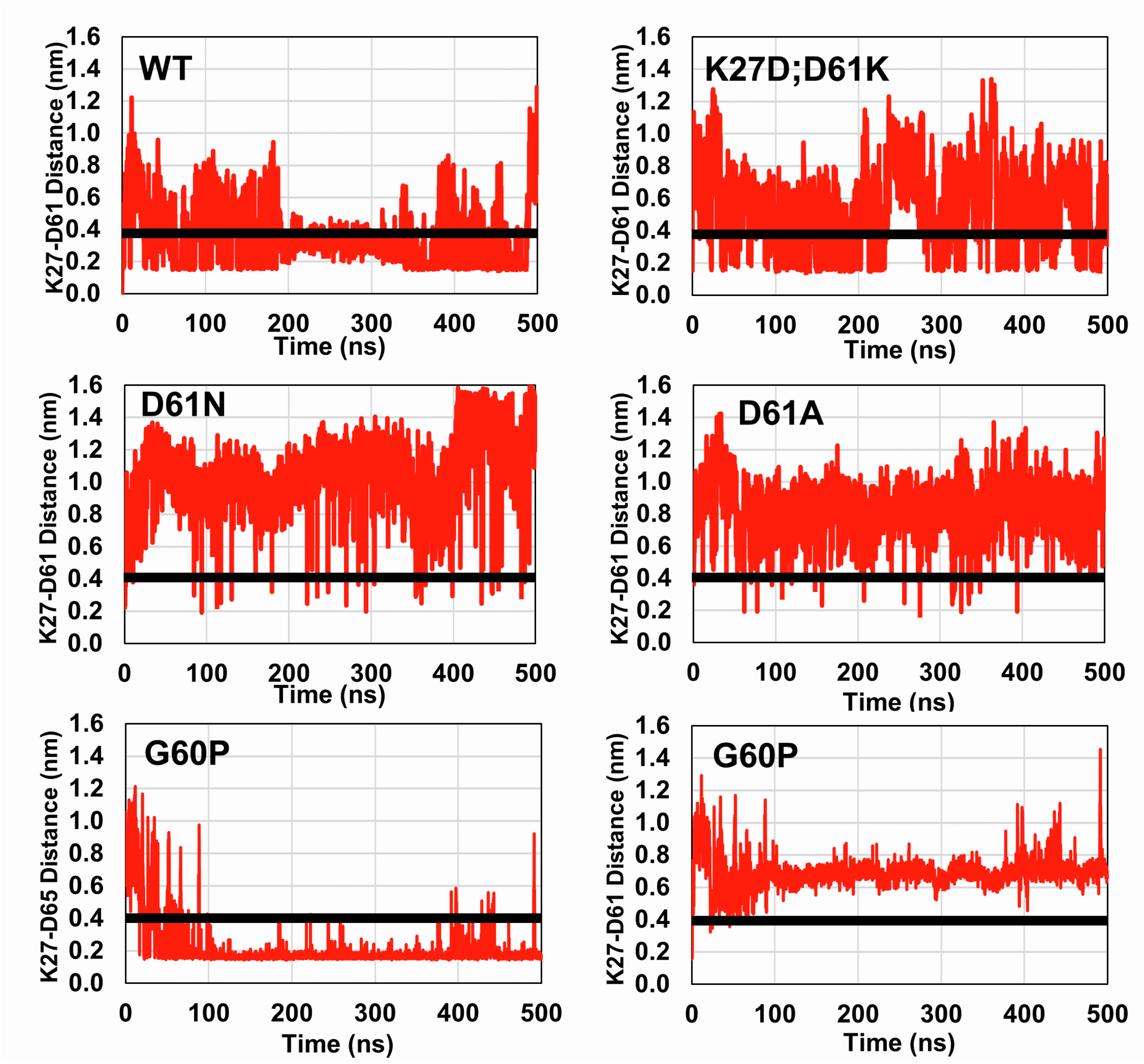

Figure S8. Monitoring open vs. closed active site loop status based on average distance between salt bridge residues 27 and 61 over time: WT (top left), p.Lys27Asp;Asp61Lys (top right), p.Asp61Asn (mid left), p.Asp61Ala (mid right), and p.Gly60Pro (bottom). Bottom right shows the average distance between an alternative salt bridge between residues 27 and 65 in the p.Gly60Pro mutant. The salt bridge formation threshold (4 Å) is shown by a black solid line.

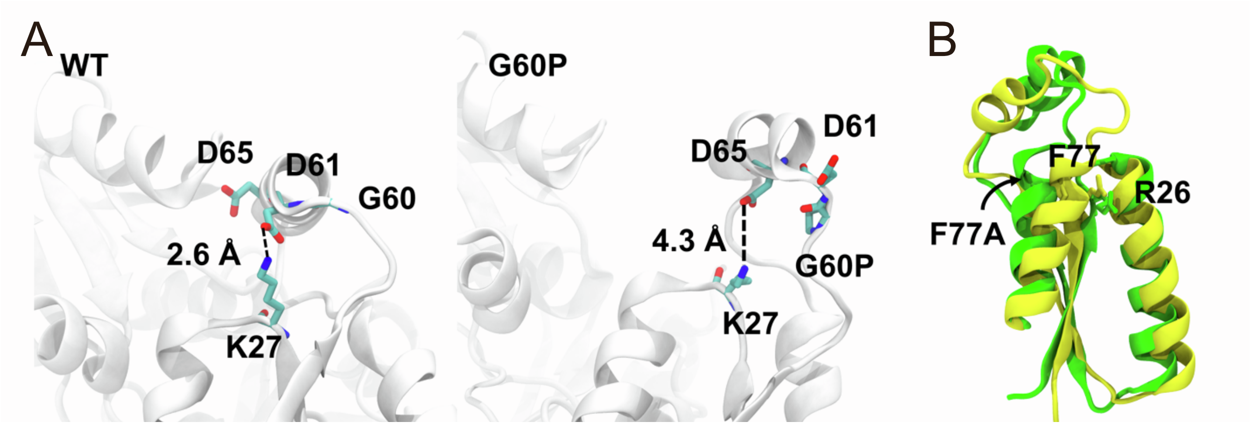

Figure S9. Modeling the effect of p.Gly60Pro and p.Phe77Ala variants on active site loop conformation. (A) Comparison of salt bridge distances (Å) in WT HMBS (left) and the p.Gly60Pro variant (right). The Asp61-Lys27 interaction is lost with the p.Gly60Pro variant, and instead replaced by a Asp65-Lys27 salt bridge that retains the active site loop in the "open" state. (B) The p.Phe77Ala variant (green) results in a dislocation of the active-site loop compared to the WT (yellow), exposing the active site.

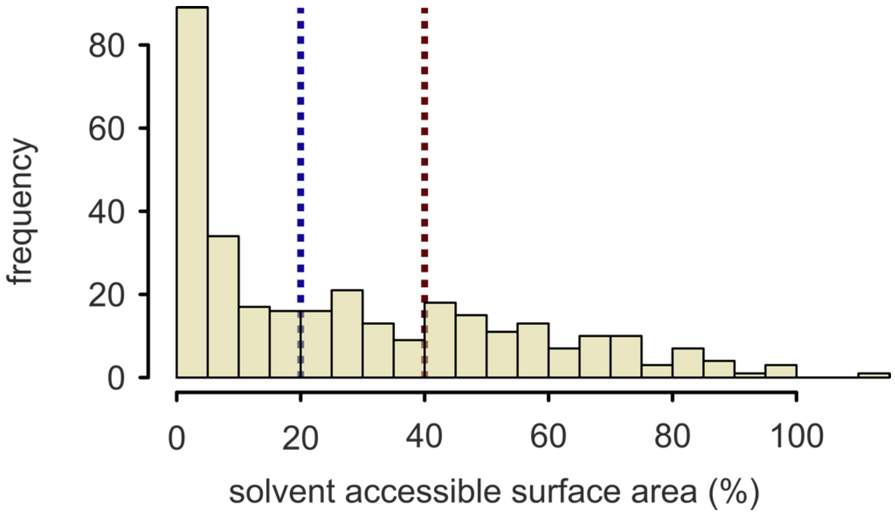

Figure S10. Distribution of solvent accessible surface area (SASA) values for HMBS residues. Residues with surface area values exceeding 40% were considered exposed, while those below 20% were classified as buried (see Methods).

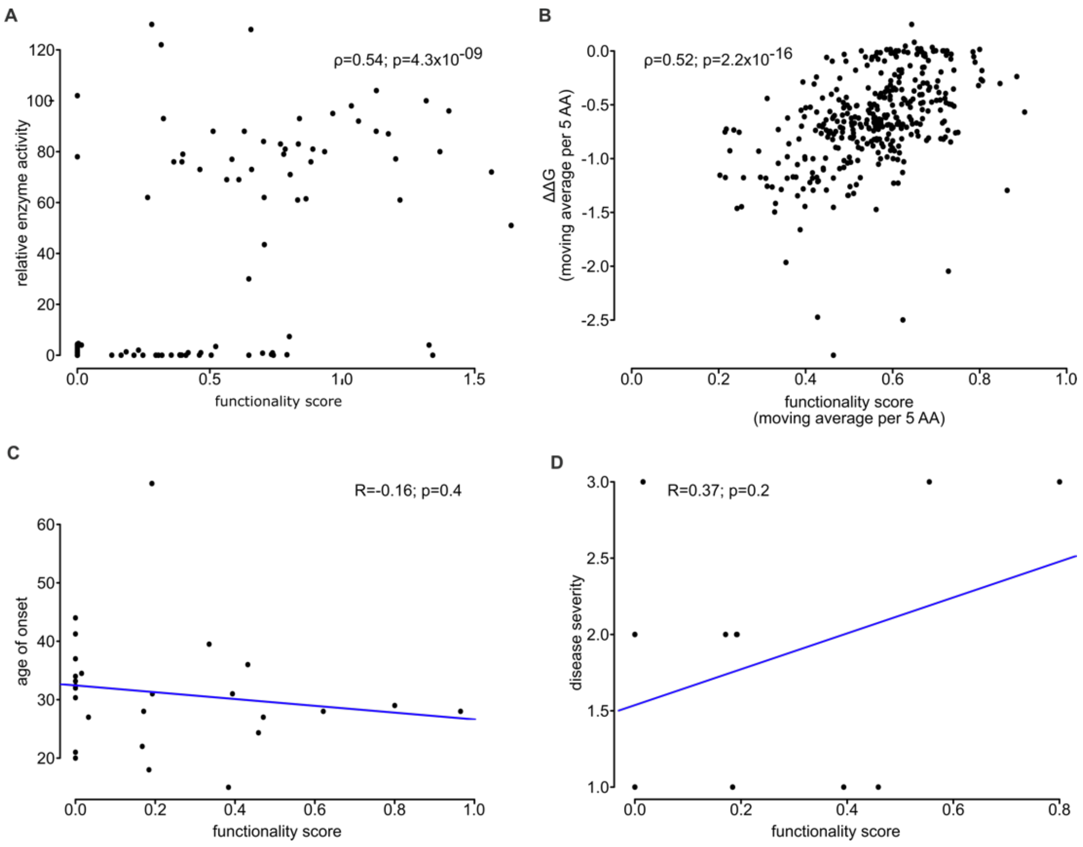

Figure S11. Correlation of functional impact scores from the combined map with other variant properties. Either Spearman's rank correlation (ρ) with significance (p) of correlation, or Pearson correlation (R) with significance (p) are shown. (A) Correlation (ϱ = 0.54, p = 4 x10^{-9}) with HMBS relative enzyme activity (variant activity divided by wild type activity). (B) Correlation (ϱ= 0.52, p = 0.2) of running medians (interval size of 5 amino acids) of the combined map scores and predicted folding free energy change (ΔΔG) values. (C) Correlation (R=-0.16; P = 0.4) with age of AIP onset. (D) Correlation (R=0.37; P = 0.2) with AIP severity scores (3 = mild disease, 2 = moderate disease, 1 = severe disease).

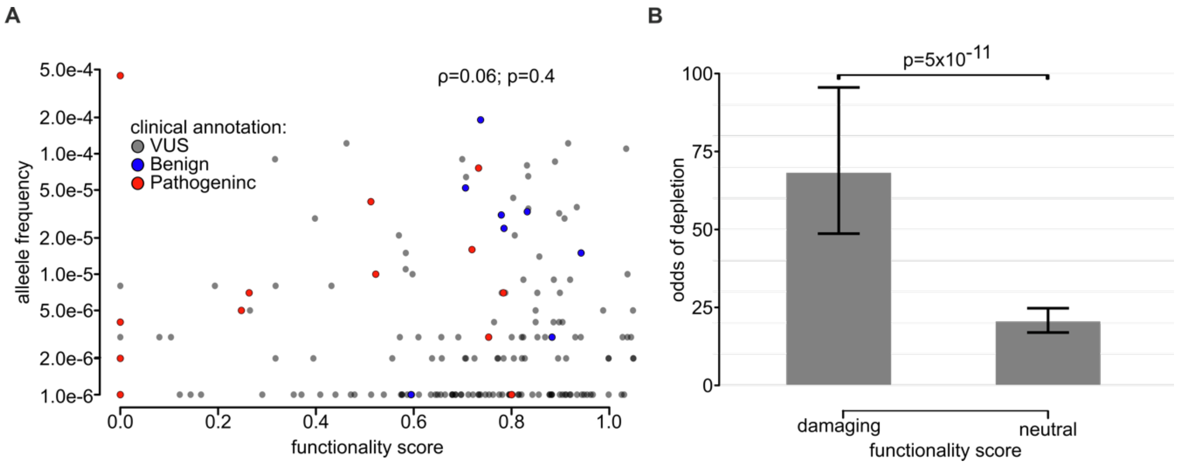

Figure S12. Depletion analysis of missense variants with neutral or damaging functional impact scores in the human population. (A) Correspondence between population allele frequency (*n* = 174) and combined HMBS map scores. For reference, annotated "pathogenic" and "benign" variants are indicated, and Spearman's rank correlation (ρ = 0.06) with significance (p = 0.4) of correlation are shown. (B) Odds ratios (OR) for the depletion of variants with neutral or damaging scores in both UK Biobank and gnomad population sequencing databases. The black lines represent 95% confidence interval (CI), and the P-value is obtained from a Fisher's exact test.

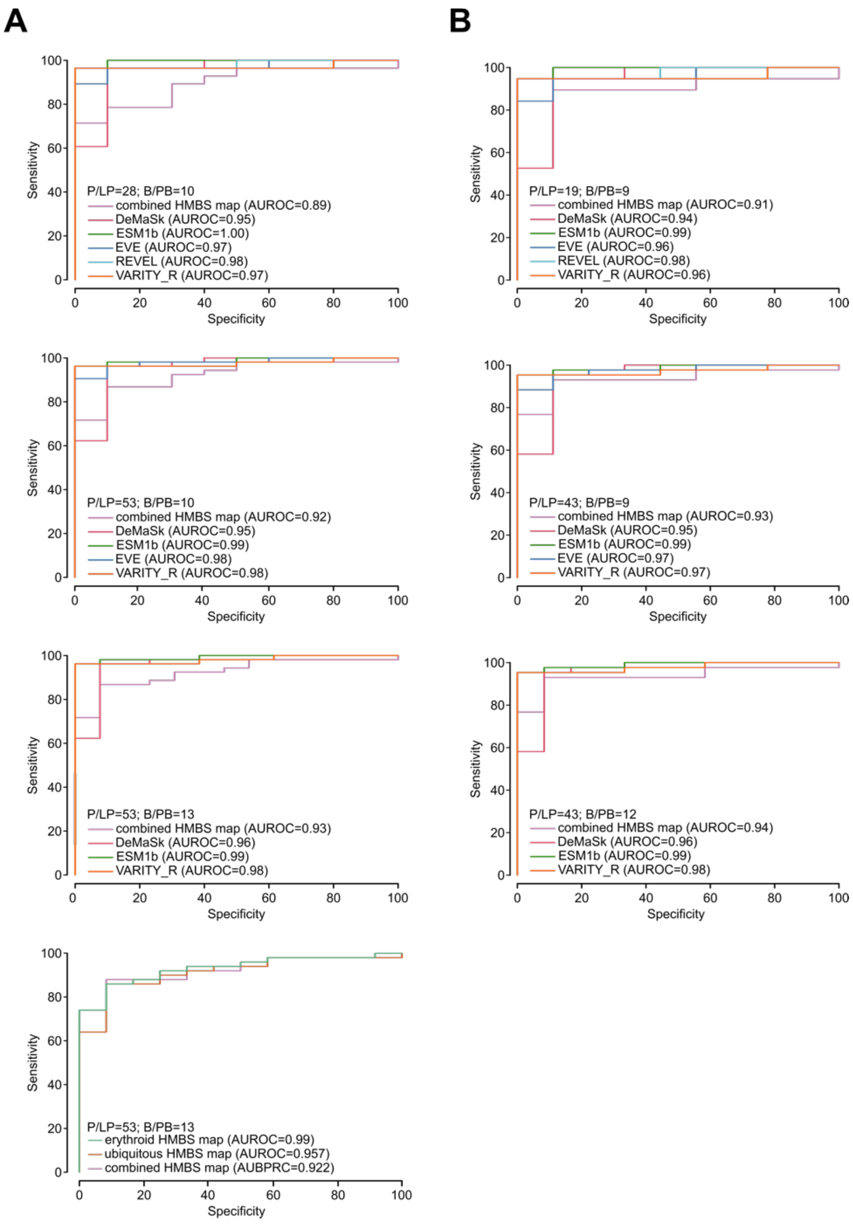

Figure S13. Comparing the combined HMBS map with computational predictors in terms of distinguishing positive from negative reference variants, as measured by receiver operating characteristic (ROC) curves for the HMBS map (pink) and computational predictors DeMaSk (red), EVE (turquoise), REVEL (blue), and VARITY_R (orange). AUROC with (A) and without (B) the inclusion of positions 160 to 215, and positions 255 and 355 are described, with positive and negative reference set sizes (P/LP and B/PB, respectively; see Methods) shown.

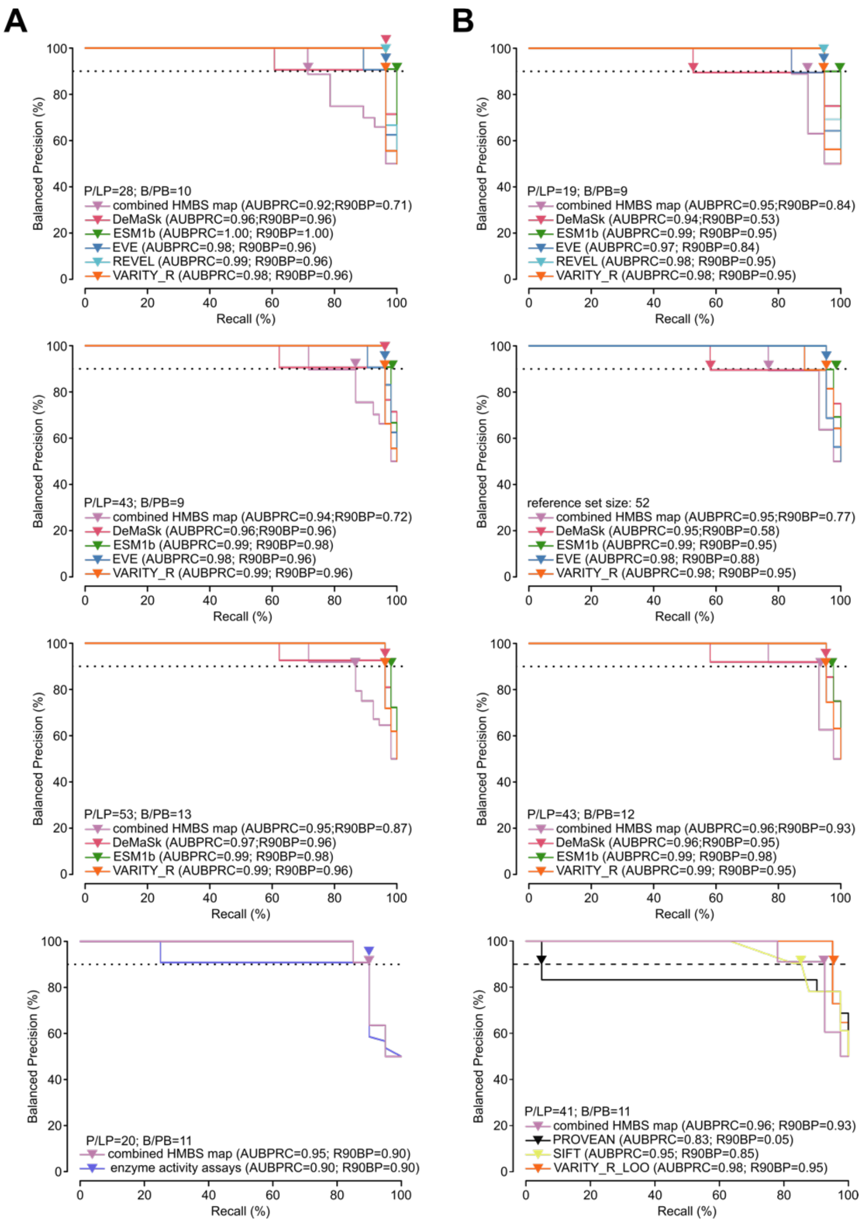

Figure S14. Comparing the combined HMBS map with computational predictors and biochemical assays in terms of distinguishing positive from negative reference variants, as measured by balanced precision recall curves. Here we evaluate precision (fraction of variants scoring below each threshold functional impact score that are in the positive reference set containing pathogenic variants) vs recall (fraction of positive reference variants with functionality scores below threshold). Precision has been transformed to reflect performance in a balanced test setting where positive and negative sets contain the same number of variants. Balanced precision-recall curves are shown for the combined map (pink), relative enzymatic activity measurements (purple) and computational predictors; DeMaSk (red), EVE (turquoise), PROVEAN (black), REVEL (blue), SIFT (yellow), and VARITY_R (orange). Performance is evaluated with (A), and without (B), the inclusion of positions 160 to 215, and positions 255 and 355, and described in terms of area under the balanced precision vs recall curve (AUBPRC) and recall at a balanced precision of 90% (R90BP). Positive and negative reference set sizes (P/LP and B/PB, respectively; see Methods) are indicated.

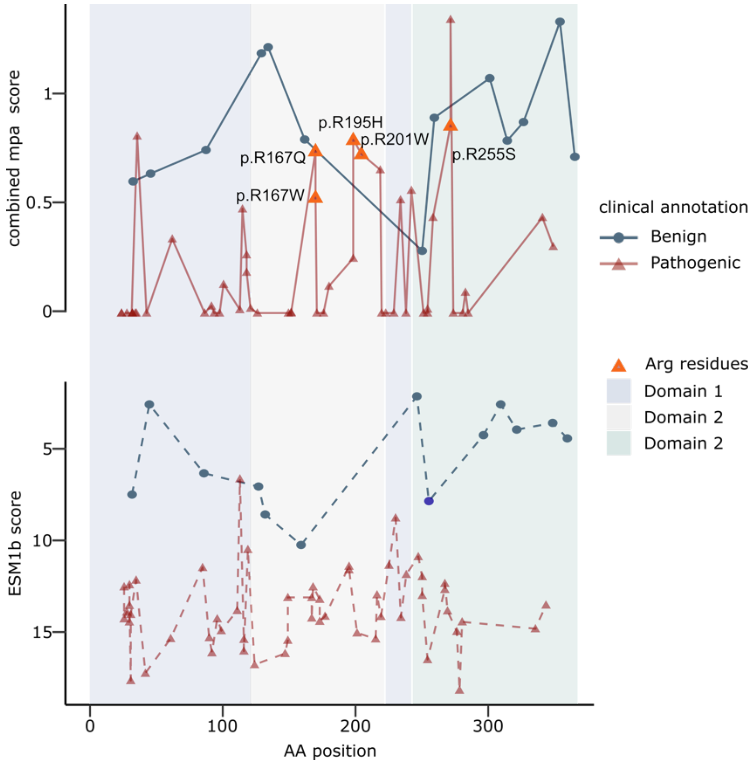

Figure S15. Correspondence of our combined map scores and those of the computational predictor ESM-1b to clinical variant annotations. Plotted values are combined map (solid line) and computational predictor ESM-1b (dotted line) scores as part of the positive (red) or negative (blue) reference variant sets. Substitutions (orange Δ) of arginine residues in positions 167, 195, and 201, that correspond to a specific limitation of our assay are highlighted. For reference, Domains 1, 2, and 3 are highlighted in light blue, gray, and green, respectively.

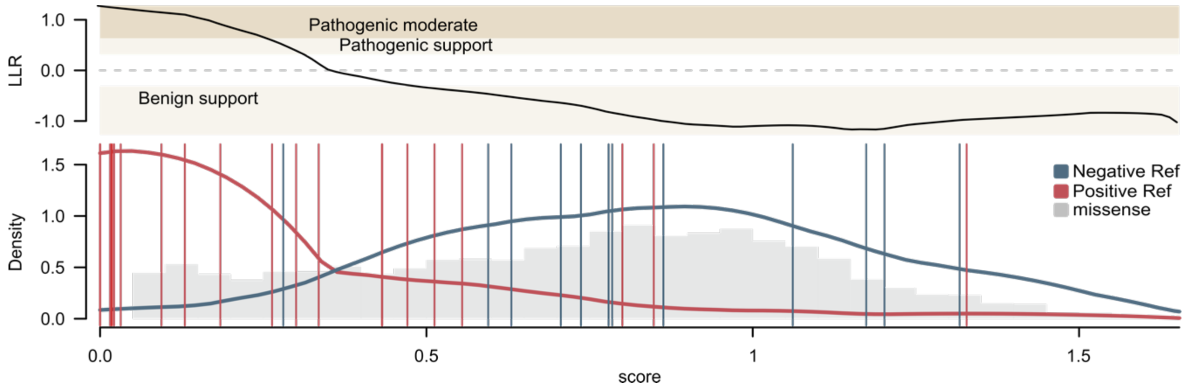

Figure S16. Transformation functions represent variant effects in terms of the strength of evidence for and against pathogenicity, that is, a log-likelihood ratio (LLR) of pathogenicity. The functions (top) express the log ratio between the likelihood of observing a given score in the score distribution of the positive reference (red) set as opposed to that of the negative reference set (blue). Gray histogram bars show the distribution of missense variants for comparison.

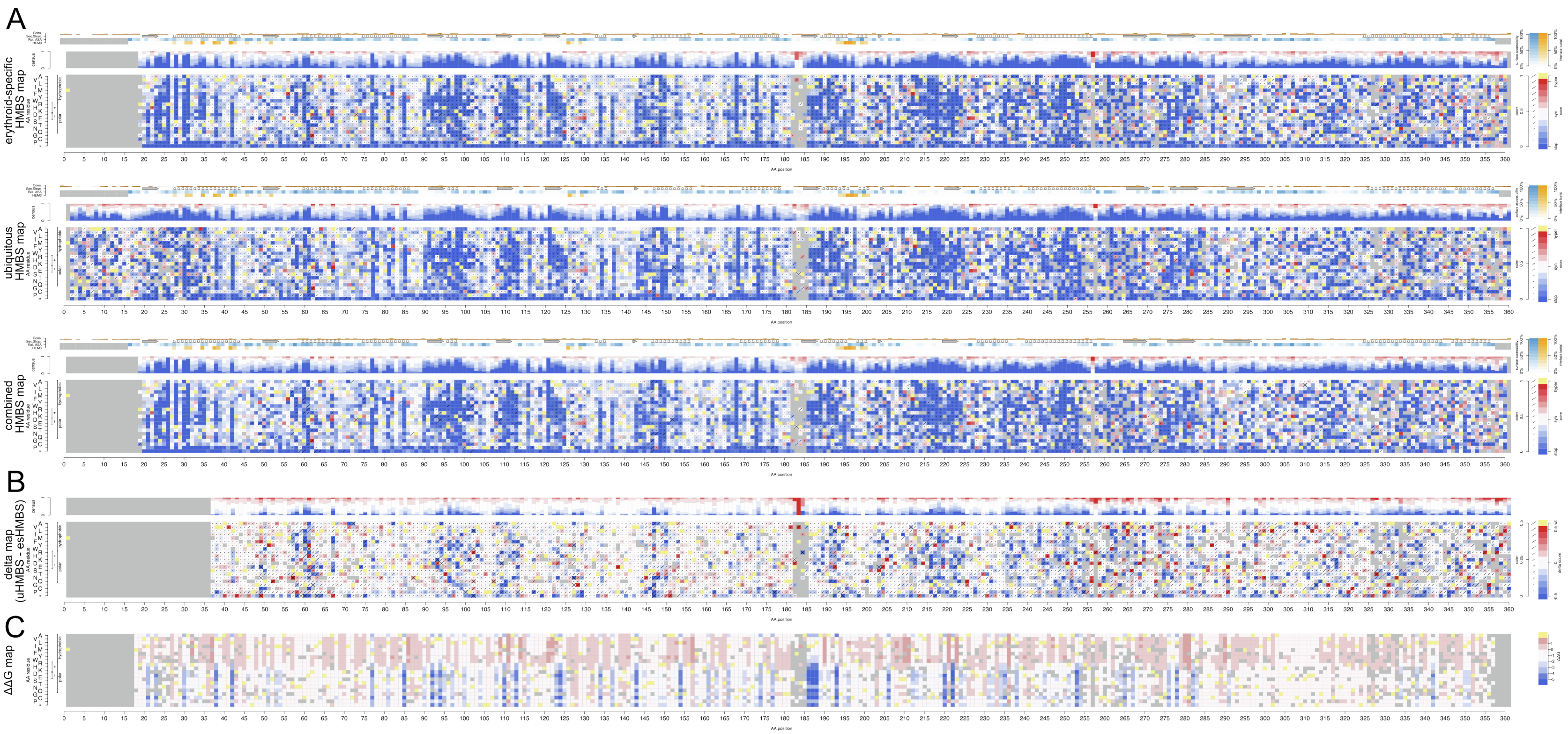

Figure S17. Full-sized HMBS variant effect maps. (A) Complete functional maps for erythroid-specific and ubiquitous HMBS isoforms, and the weighted average score values. Colors and labels are as in Figure 2. (B) Delta map, measuring the difference in functional impact between ubiquitous and erythroid-specific isoforms. Substitutions were coloured red if the score is positive and blue if negative. (C) Map of predicted stability (ΔΔG) effects for HMBS variants. Box color either indicates the wild-type residue (yellow), a stabilizing (red), destabilizing, or neutral variant (white), or missing data (gray).

Supplemental tables

Model	ΔAIC relative to best model
Penalize enhancing mutations	0
Cap score of enhancing mutations at wild type level	24.1
Enhancing mutations are beneficial	38.0

Table S1. Comparing different models for effects of activity enhancing mutations.

System	RMSD (nm)
Apo-enzyme WT	0.21 (0.02)
Apo-enzyme G60P	0.33 (0.05)
Apo-enzyme D61N	0.19 (0.04)
Apo-enzyme D61A	0.19 (0.04)
Apo-enzyme K27D D61K	0.31 (0.03)
Apo-enzyme R26P	0.27 (0.04)
ES2 WT	0.23 (0.03)
ES2 E250R	0.22 (0.02)
ES2 G346P	0.23 (0.02)

Table S2. Average RMSD (standard deviation in parentheses) of backbone atoms for the wild-type (WT) or enzyme substrate complex 2 (ES2) structures.

	DPM-PBG		
**Residue**	**WT**	**E250R**	**G346P**
R149	100	82	87
R150	100	85	100
G218	100	-	95
S147	99	57	65
S96	99	55	65
A189	98	56	73
R173	98	95	42
T102	100	58	70
L148	72	-	-
K98	100	100	100
S146	68	99	54
R195	57	76	50
R117	-	93	86
T145	68	85	38
D99*	94, 80	20, 4	7, 6

*Numbers correspond to hydrogen bonds with the 4th (second PBG) and 3rd (first PBG) pyrrole ring, respectively.

Table S3. Hydrogen bond occupancy (%) between HMBS important residues and the substrate in different systems. For residues with more than one interaction with PBG, only the more persistent interaction is reported.

Backbone Hydrogen Bonds		Occupancy (%) G346P	WT
L342 (O)	K345 (NH)	80	-
L342 (O)	A347 (NH)	-	87
L342 (O)	G346 (NH)	-	57
L342 (NH)	L338 (O)	99	100
L343(O)	G346 (O)	-	46
L343 (NH)	A339 (O)	92	97
S344 (NH)	L341 (O)	62	20
S344 (NH)	N340 (O)	-	78
K345 (O)	A347 (NH)	91	-
K345 (NH)	L342 (O)	80	-
K345 (NH)	L341 (O)	-	90
G346 (O)	N349 (NH)	-	55
G346 (NH)	L342 (O)	-	57
G346 (NH)	L343 (O)	-	46
P346 (O)	N349 (NH)	47	-
P346 (O)	I350 (NH)	32	-
A347 (O)	I350 (NH)	49	65
A347 (O)	L351 (NH)	92	87
A347 (NH)	K345 (O)	91	-
A347 (NH)	L342 (O)	-	87

Table S4. Hydrogen bond occupancy (%) in the C-terminal helix region for WT and the G346P variant.

G346P		
**Hydrogen Bond**		**Occupancy (%)**
R355 (NH1)	G259 (O)	82
R355 (NH2)	L257 (O)	63
R355 (NH22)	D352 (Oδ2)	59
R355 (NH21)	D352 (Oδ1)	32
**WT**		
**Hydrogen Bond**		**Occupancy (%)**
R355 (NH1)	D352 (Oδ1)	19
R355 (NH)	D352 (O)	16

Table S5. Hydrogen bond occupancy (%) for R355 in WT and the G346P variant.

	WT	K27D D61K	D61N	D61A	G60P	R26P
R26-D61	7.6	8.6	12	11	14	13
K27-D61	3.5	5.4	11	8.5	6.9	10
G60-Q34	17	18	20	20	21	20
G60-R26	9.1	8.9	10	11	13	12

Table S6. Average distance (Å) between selected residue pairs in WT and variants.

Supplemental references

1. Frazer, J., Notin, P., Dias, M., Gomez, A., Min, J.K., Brock, K., Gal, Y., and Marks, D.S. (2021). Disease variant prediction with deep generative models of evolutionary data. Nature *599*, 91–95.

2. Ioannidis, N.M., Rothstein, J.H., Pejaver, V., Middha, S., McDonnell, S.K., Baheti, S., Musolf, A., Li, Q., Holzinger, E., Karyadi, D., et al. (2016). REVEL: An Ensemble Method for Predicting the Pathogenicity of Rare Missense Variants. Am. J. Hum. Genet. *99*, 877–885.

3. Lenglet, H., Schmitt, C., Grange, T., Manceau, H., Karboul, N., Bouchet-Crivat, F., Robreau, A.-M., Nicolas, G., Lamoril, J., Simonin, S., et al. (2018). From a dominant to an oligogenic model of inheritance with environmental modifiers in acute intermittent porphyria. Hum. Mol. Genet. *27*, 1164–1173. PMID: 29360981

4. Bung, N., Roy, A., Chen, B., Das, D., Pradhan, M., Yasuda, M., New, M.I., Desnick, R.J., and Bulusu, G. (2018). Human hydroxymethylbilane synthase: Molecular dynamics of the pyrrole chain elongation identifies step-specific residues that cause AIP. Proc. Natl. Acad. Sci. U. S. A. *115*, E4071–E4080. PMID: 29632172

5. Bustad, H.J., Vorland, M., Rønneseth, E., Sandberg, S., Martinez, A., and Toska, K. (2013). Conformational stability and activity analysis of two hydroxymethylbilane synthase mutants, K132N and V215E, with different phenotypic association with acute intermittent porphyria. Biosci. Rep. *33*, e00056. PMID: 23815679

6. Chen, B., Solis-Villa, C., Erwin, A.L., Balwani, M., Nazarenko, I., Phillips, J.D., Desnick, R.J., and Yasuda, M. (2019). Identification and characterization of 40 novel hydroxymethylbilane synthase mutations that cause acute intermittent porphyria. J. Inherit. Metab. Dis. *42*, 186–194. PMID: 30740734

7. Bustad, H.J., Kallio, J.P., Vorland, M., Fiorentino, V., Sandberg, S., Schmitt, C., Aarsand, A.K., and Martinez, A. (2021). Acute Intermittent Porphyria: An Overview of Therapy Developments and Future Perspectives Focusing on Stabilisation of HMBS and Proteostasis Regulators. Int. J. Mol. Sci. *22*, 675. PMID: 33445488

8. Chen, B., Solis-Villa, C., Hakenberg, J., Qiao, W., Srinivasan, R.R., Yasuda, M., Balwani, M., Doheny, D., Peter, I., Chen, R., et al. (2016). Acute Intermittent Porphyria: Predicted Pathogenicity of HMBS Variants Indicates Extremely Low Penetrance of the Autosomal Dominant Disease. Hum. Mutat. *37*, 1215–1222. PMID: 27539938
